# Integrated metabolome and microbiome analysis reveals the effect of rumen-protected sulfur-containing amino acids on the meat quality of Tibetan sheep meat

**DOI:** 10.3389/fmicb.2024.1345388

**Published:** 2024-02-08

**Authors:** JiQian Liu, Lijuan Han, Shengzhen Hou, Linsheng Gui, Zhenzhen Yuan, Shengnan Sun, Zhiyou Wang, Baochun Yang

**Affiliations:** College of Agriculture and Animal Husbandry, Qinghai University, Xining, China

**Keywords:** Tibetan sheep, rumen protected sulfur-containing amino acids (RPSAA), meat quality, metabolomics, gastrointestinal microbiota

## Abstract

**Introduction:**

This study investigated the effects of rumen-protected sulfur-containing amino acids (RPSAA) on the rumen and jejunal microbiota as well as on the metabolites and meat quality of the *longissimus lumborum* (LL) in Tibetan sheep.

**Methods:**

By combining 16S rDNA sequencing with UHPLC-Q-TOF MS and Pearson correlation analysis, the relationship between gastrointestinal microbiota, muscle metabolites and meat quality was identified.

**Results:**

The results showed that feeding RPSAA can increase the carcass weight, abdominal fat thickness (AP-2 group), and back fat thickness (AP-2 and AP-3 group) of Tibetan sheep. The water holding capacity (WHC), texture, and shear force (SF) of LL in the two groups also increased although the fatty acids content and brightness (L*) value significantly decreased in the AP-2 group. Metabolomics and correlation analysis further showed that RPSAA could significantly influence the metabolites in purine metabolism, thereby affecting L* and SF. In addition, RPSAA was beneficial for the fermentation of the rumen and jejunum. In both groups, the abundance of *Prevotella 1*, *Lachnospiraceae NK3A20 group*, *Prevotella UCG-003*, *Lachnospiraceae ND3007 group* in the rumen as well as the abundance of *Eubacterium nodatum group* and *Mogibacterium group* in the jejunum increased. In contrast, that of *Turicibacter* pathogens in the jejunum was reduced. The above microorganisms could regulate meat quality by regulating the metabolites (inosine, hypoxanthine, linoleic acid, palmitic acid, etc.) in purine and fatty acids metabolism.

**Discussion:**

Overall, reducing the levels of crude proteins in the diet and feeding RPSAA is likely to improve the carcass quality of Tibetan sheep, with the addition of RPMET (AP-2) yielding the best edible quality, possibly due to its ability to influence the gastrointestinal microbiota to subsequently regulate muscle metabolites.

## Introduction

Tibetan sheep, a unique local breed in the Qinghai Tibet region of China, exhibits great adaptability to harsh environments, including cold weather, hypoxia and nutrient scarcity (Su et al., 2022). Studies have found that Tibetan sheep meat possesses desirable qualities in terms of its fresh and tender taste, its high protein but low fat content, its diverse types of amino acids as well as its rich aroma (Zhang et al., 2022a). These attributes actually fulfil the dietary requirements of modern consumers. However, in recent years, ecological factors, along with the limited availability of grassland areas, have gradually shifted the grazing practices in animal husbandry to housed or semi-housed feeding (Zhang et al., 2022b). As a result, this has led to an increased demand for protein feed to promote animal growth. In addition, the incomplete digestion and absorption of dietary proteins by animals increases the emission of nitrogen pollutants, thereby resulting in environmental pollution (Hristov et al., 2011) that subsequently restricts the development of animal husbandry (Qi et al., 2023). However, in an attempt to address the issue, researchers have found that supplementing animal diets with rumen-protected amino acids (RPAA) can lower the level of dietary proteins and hence, reduce nitrogen emission (Ma et al., 2010; Kamiya et al., 2021).

Sulfur containing amino acids (SAA) include methionine (MET), cysteine (CYS), cystine, taurine, etc. MET and CYS are considered to be the main SAAs because they are typical amino acids involved in protein synthesis (Brosnan and Brosnan, 2006). MET is the only sulfur-containing essential amino acid in animal growth, and it is involved in protein synthesis, methylation cycle, polyamine production, sulfur transfer pathway and other important metabolic reactions in animals (Finkelstein, 1990). CYS can be used as a precursor of important bioactive substances such as glutathione, hydrogen sulfide and taurine, thus playing antioxidant, digestive and immune roles (Blachier et al., 2020). Some studies have reported that dietary SAA supplementation has important nutritional significance for animal gastrointestinal development. Zhong et al. studied the effect of DL-Met addition on the intestinal development of young pigeons and found that 0.3% DL-Met could improve the intestinal morphology and structure of pigeons by activating Wnt/β-catenin signaling pathway (Zhong et al., 2022). Gu et al. (2021) added 20 gm/day of RPMET to the diet of dairy cows in the middle of lactation to promote rumen fermentation and increase the beneficial microorganisms such as *Acetobacter*, *saccharofermentan*, *Thermoactinomyces* and so on that promote milk synthesis. Similarly, Ding et al. (2019) found that adding 0.4% CYS to the piglet diet can improve the level of antioxidant stress metabolites in the plasma by increasing the abundance of *Lactobacillus* and *pseudobutyrivibrio* in the intestine. Ni et al. (2021) showed that providing 0.4 and 0.5% of CYS to the piglet mother would reduce the level of *Firmicutes* in the piglet colon and increase the level of *Bacteroides*. This change may affect the lipid metabolism of piglets. With further research, it was found that adding SAA also had an impact on meat quality. Lebret et al. (2018) found that adding 1.1% MET to the pig diet can improve the final pH value of pork and reduce drip loss, brightness and hue. Similarly, adding a high level of MET (21.36%) to the diet of arbor acres broilers also improved the pH value of broilers and significantly reduced the brightness and cooking loss (Wen et al., 2017). On the other hand, Conde Aguilera et al. found that providing sufficient SAA (0.46% MET and 0.70% MET+CYS) to piglets would increase the protein and water content of longissimus muscle of pig waist and reduce the content of fat, ash and total amino acids (Conde-Aguilera et al., 2010). The above results show that SAA supplementation in the diet will have an impact on gastrointestinal microorganisms and meat quality of livestock and poultry, However, there is a limited body of research examining the impact of dietary supplementation with MET and CYS on the gastrointestinal tract and meat quality of Tibetan sheep.

The rapid development of metabolomics made it possible for scientists to delve deeper into research on metabolites and meat quality. For example, studies by Zhang et al. found that changes in the metabolic pathways of amino acids, lipids and carbohydrates in muscles altered metabolite levels that subsequently influenced the tenderness and flavor of meat (Zhang et al., 2022a,b). Similarly, MET, LYS and CYS are essential bioactive molecules that participate in key metabolic pathways in the body (Watanabe et al., 2020; Gondret et al., 2021), and as such, changes in their levels may also exert similar effects. In addition, rumen microorganisms are of great significance in animal nutrition digestion, absorption and metabolism (Zhang et al., 2020). Studies from Ding and Burrin showed that adding amino acids to animal diets can provide energy for the small intestine, increase the abundance of intestinal microbiota as well as maintain intestinal integrity (Burrin and Reeds, 1997; Ding et al., 2019). In this context, studies by Ma et al. (2023) revealed that feed additives could influence muscle metabolites and improve meat quality by altering the composition of rumen microorganisms in Tibetan sheep. In a similar vein, Zhiwei (Zhao et al., 2021) found that supplementing the diet of lactating yaks with RPMET and RPLYS could alter the composition of their rumen bacteria, influence the rumen fermentation environment and metabolism and ultimately alter the synthesis of metabolites in milk. Based on the above, the current study hypothesized that RPLYS, RPMET and RPCYS have the potential to modulate the gastrointestinal microbiota of Tibetan sheep, thereby affecting muscle metabolites and meat quality.

At present, the price of protein feed is becoming increasingly expensive, and high protein feed will increase animal nitrogen excretion and cause serious environmental pollution (Wei et al., 2023). In order to save the feeding cost, we need to reduce the protein level of feed, which will lead to the reduction of growth performance of livestock and poultry due to insufficient nutrition, and have a negative impact on meat quality (Zhang et al., 2024). The study found that maintaining a better amino acid balance at a low protein level can improve the utilization rate of protein raw materials, effectively relieve the current pressure of resource supply and demand, and achieve a win-win situation in productivity and environmental benefits (Liu et al., 2024). Therefore, this work applied 16S rDNA and metabolomics techniques to investigate the effects of rumen-protected sulfur-containing amino acids (RPSAA) on the gastrointestinal microbiota and muscle metabolites of Tibetan sheep. Correlation analysis was subsequently used to reveal the relationship between Tibetan sheep meat quality, gastrointestinal microbiota and muscle metabolism, thereby elucidating the mechanism through which RPSAA influences the meat quality. It is expected that the results of this study will be of significance to determine the optimal amount and specific types of RPAA to be added to Tibetan sheep feed, hence helping to reduce the level of feed protein, save feed protein resources, reduce feeding costs, while reducing environmental pollution and promoting the sustainable development of animal husbandry in the Qinghai Province.

## Materials and methods

### Experimental animals, slaughter and collection of meat samples

Ninety healthy female Tibetan sheep of 2–3-month-old and with an initial weight of 13.5 kg were randomly divided into three groups (*n* = 30). For the first group (AP-1, ordinary amino acid group), regular lysine and regular methionine were added to the diet in a ratio of 3 kg: 1 kg. The diet of the second group (AP-2, RPAA group) was supplemented with rumen-protected lysine (RPLYS) and rumen-protected methionine (RPMET) in a ratio of 1.5 kg: 0.5 kg group, while for the third one (AP-3, RPCYS group), RPLYS, RPMET and rumen-protected cysteine (RPCYS) were added in a ratio of 1.5 kg: 0.25 kg: 0.25 kg. The latter two, namely the AP-2 and AP-3 groups, were also referred to as the RPSAA group. All animals were fed separately at 8:00 am and 18:00 pm and following a 7-day adaptation period, they were subjected to the experimental conditions for 120 days during which they had free access to feed and water. The composition and nutritional level of the diets are shown in Supplementary Table S1.

At the end of the experiment, the test animals were transported to a nearby commercial slaughterhouse where, after 24 h of fasting and 2 h of water deprivation, they were slaughtered according to established animal welfare procedures. The *longissimus lumborum* (LL) between the 9th and 11th ribs was then collected and after removing the fat and fascia, it was kept in dry ice until transport to the laboratory where it was stored in a refrigerator at −80°C. In addition, the rumen and jejunum were cut open to collect the contents which were subsequently filtered through four layers of sterile gauze. The pH value of 50 mL of rumen fluid was then immediately measured. The rumen fluid and jejunum contents were also divided between frozen storage tubes and stored in a liquid nitrogen tank before being transported to the laboratory where they were stored at −80°C.

### Analysis of the carcass quality

To analyze the carcass quality, the body size index of Tibetan sheep was first measured before slaughter. The carcass weight (CW) – the weight of the slaughtered Tibetan sheep after full bloodletting – was then determined. The fur, head, hoof, tail and viscera (kidney and suet) were also removed and weighed before measuring the following: the body height (BH) which was the vertical distance from the highest point of the jaguar to the ground, the body length (BL) which was the linear distance from the front end of the scapula to the back end of the ischium and the chest circumference (CC) which was the length of the posterior edge of the scapula around the chest vertically. Furthermore, during the process of carcass segmentation, cross sections were made between the 12th and 13th ribs, with the cross section of the eye muscles depicted with sulfuric acid paper. This was followed by measurements of the fat thickness using a vernier caliper. In particular, the rib fat thickness (RFT – located 110 mm from the midline of the spine), the back fat thickness (BFT – located directly above the middle of the eye muscles) and the abdominal fat thickness (AFT – located 127 mm from the midline of the spine) were determined.

### Analysis of meat quality

#### Meat edible quality and nutritional component analysis

After slaughter, part of the LL of Tibetan sheep was used for colorimetric measurements. This involved using a PHSJ-4A colorimeter (Shanghai, China) that had been calibrated with a black and white standard plate to measure brightness (L^*^), redness (a^*^), and yellowness (b^*^). The pH of the meat after 45 min (pH_45min_) and 24 h (pH_24h_) were also determined by inserting the probe of a calibrated pH meter (PHSJ-6 L, Shanghai, China) into a previously marked area on the meat surface.

Meat samples stored at −80°C were weighed before (W1) and after thawing at 4°C for 12 h (W2) to determine the thawing loss as follows: thawing loss (%) = (W1-W2)/W1. After thawing meat samples, the surface moisture was dried and the weight (M1) was recorded. They were then kept in a steaming bag and heated for 20 min in a water bath at 85°C. The samples were subsequently dried and weighed (M2) to determine the cooking loss (%) as (M1-M2)/M1. In addition, weighed meat samples (Q1) were placed in a steaming bag and heated in water bath at 85°C for 40 min before recording the new weight (Q2). From the readings, the cooked meat rate (%) was then calculated as Q2/Q1. The rate of water loss was also measured using the MAEC-18 series hydraulic tester (Nanjing, China). This involved applying a pressure of 350 N to a meat block (N1) of 1 cm × 1 cm × 1 cm. Based on the amount of water lost of the meat block after applying pressure (N2), the rate of water loss rate (%) was then calculated as N2/N1. The shear force (SF) was determined using the MAQC-12 shear force instrument (Nanjing, China). In this case, the meat samples were first heated at 80°C in a water bath until the center temperature reached 70°C. Readings were then taken using 1 cm × 1 cm × 3 cm long strips, with the muscle fibers being perpendicular to the cutting tool during the measurement. The final measurements involved assessing the texture properties, such as the hardness, elasticity, stickiness, adhesiveness, cohesion and chewiness of the meat, using a texture analyzer (Universal TA, Shanghai, China).

The nutritional composition of the meat was also determined using recognized methods of the Association of Analytical Chemists (AOAC, 2000), while the moisture content was measured by drying the samples in an oven at 103 ± 2°C. In addition, the protein and fat content were determined using the Kjeldahl method and the Soxhlet extraction method, respectively. For all samples, triplicate measurements were taken in parallel.

#### Analysis of the amino acids and fatty acids

The composition of amino acids was determined with an Agilent 1,290 Infinity LC ultra-high performance liquid chromatography (Beijing, China) and a 5,500 QTRAP mass spectrometer (Beijing, China). For this purpose, 60 mg of meat sample was mixed with a methanol-acetonitrile mixture in a volume ratio of 1:1. This was followed by the addition of 50 ul of an internal standard mixture (16 isotope internal standards) and after vortex mixing, ultrasound was applied for 30 min (cycling twice). Finally, the protein was precipitated by allowing the mixture to stand at −20°C. The supernatant was subsequently centrifuged and freeze-dried prior to storage at −80°C. The parameters for high-performance liquid chromatography and mass spectrometry were as described by Zhang et al. (2022b). Basically, this involved an injection volume of 1 ul, a column temperature of 40°C and a flow rate of 250 ul/min for the chromatographic separation. In addition, the mobile phase A consisted of 25 mM ammonium formate and 0.08% of aqueous FA solution, while mobile phase B consisted of 0.1% FA acetonitrile. Similarly, the mass spectrometry was performed under the following conditions: a source temperature of 500°C, ion Source Gas1 (Gas1): 40, Ion Source Gas2 (Gas2): 40, Curtain gas (CUR): 30 and an ionSapary Voltage Floating (ISVF) of 5,500 V. The ion pairs being tested were then detected using the MRM mode.

To determine the fatty acids composition, GC–MS was performed as previously described (Zhang et al., 2022b). Lipids were first extracted from 50 mg of accurately-weighed Tibetan sheep meat sample using chloroform-methanol (2:1, v/v) solution. Standard solutions of fatty acid methyl esters were then prepared using n-hexane prior to gas chromatography under the following conditions: a Thermo TG-FAME capillary column (50 m * 0.25 mm ID * 0.20 μm) for the separation, a sample inlet temperature of 250°C, an ion source temperature of 300°C, a transmission line temperature of 280°C and helium at a flow rate of 0.63 mL/min as the carrier. Furthermore, the programmed heating conditions consisted of an initial temperature of 80°C that was maintained for 1 min. It was then raised to 160°C at a rate of 20°C/min and maintained for 1.5 min before being raised to 196°C at a rate of 3°C/min. This temperature, maintained for 8.5 min, was subsequently raised to 250°C at a rate of 20°C/min and maintained for 3 min. For mass spectrometry analysis, a Thermo Trace 1,300/TSQ 9000 gas-mass spectrometry (Shanghai, China) was used with an electron bombardment ionization (EI) source and a SIM scanning method. In addition, an injection port temperature of 280°C, an ion source temperature of 230°C, a transmission line temperature of 250°C and an electron energy of 70 eV were applied. Finally, draw the calibration curve (Supplementary Table S2) and calculate the content of fatty acids in the sample.

### Analysis of non-target metabolites in meat samples

Non-target metabolites in the meat samples were identified using ultra-high performance liquid chromatography tandem time of flight mass spectrometry (UHPLC-Q-TOF MS). An extraction solution consisting of methanol/acetonitrile/water solution (2:2:1, v/v) was first added to 100 mg of meat samples that had been thawed at 4°C. The mixture was then vortexed and after applying ultrasound, it was centrifuged, with the resulting supernatant subsequently vacuum dried. A 50-ul mixture of acetonitrile and water (1:1, v/v) was then added prior to vortex mixing and centrifugation. The final supernatant was analyzed with an Agilent 1,290 Infinity LC Ultra-High Performance Liquid Chromatography (UHPLC) (Beijing, China) HILIC column using a column temperature of 25°C and a sample injection volume of 2 ul. Elution was achieved using mobile phase A (water±25 mM ammonium acetate ± 25 mM ammonia) and mobile phase B (acetonitrile) at a flow rate of 0.5 mL/min, with the elution gradient being as described by Zhang et al. (2022a).

A Triple TOF 6600 mass spectrometer (AB SCIEX) (Beijing, China) was used for mass spectrometry analysis, with the positive and negative ion modes of electric spray ionization (ESI) used for detection. The ESI source conditions were set as follows: atomizing gas auxiliary heating gas 1 (Gas1): 60, auxiliary heating gas 2 (Gas2): 60, curtain gas (CUR): 30 psi, ion source temperature: 600°C, spray voltage (ISVF) ± 5,500 V (positive and negative modes).

After all measurements, data processing, including peak alignment, retention time correction and peak area extraction, was performed with the XCMS software. The results were subsequently analyzed using SIMCA software (version 14.0) to determine differences between the groups. In particular, functional annotation of differential metabolites and the identification of their corresponding metabolic pathway were performed using the Kyoto Encyclopedia of Genes and Genomes database (KEGG, www.genome.jp/kegg).

### The composition of gastrointestinal microbiota

A 50-ml centrifuge tube was filled with filtered gastric juice to determine its pH. In addition, for the gastric juice and jejunal contents, ammoniacal nitrogen measurements were obtained using alkaline sodium hypochlorite phenol spectrophotometry, while gas chromatography allowed the identification of their short chain fatty acids (SCFAs).

To determine the gastrointestinal microbiota, gastric juice and jejunal content were collected in a cryopreservation tube and immediately frozen in liquid nitrogen for storage at −80°C. Amplification and sequencing of the V3-V4 variable region were then performed on the Illumina NovaSeq 6,000 sequencing platform as described by Zhang et al. (2022a). Basically, DNA was extracted from thawed samples before generating amplicons by PCR. In this case, the resulting PCR products were detected by 2% agarose gel electrophoresis, with the AxyPrepDNA gel recovery kit (AXYGEN Company) and the QuantiFluor^™^—ST Blue Fluorescence Quantitative System (Promega Company) subsequently used for recovery and quantification. The PCR products were then mixed and purified before library preparation using the NEB Next^®^ Ultra^™^ DNA Library Prep Kit. The quality of generated libraries was eventually assessed with an Agilent Bioanalyzer 2,100 and Qubit prior to sequencing.

After sequencing, paired end reads were merged with FLASH software. Sequences were then clustered into OTUs at a similarity level of ≥97% using UPARSE software, with the annotation and classification of the representative sequence in each OTU subsequently performed using an RDP classifier. Finally, the results were processed to analyze various indicators.

### Statistical analysis

SPSS 25.0 was used to analyze the carcass quality, muscle quality (edible quality, nutrition quality, AA, FA), muscle metabolites and gastrointestinal microbiota of Tibetan sheep. All data were expressed as mean ± standard error (SE), with *p-*values *<0.05* indicating significant differences. Pearson correlation was also used to analyze the relationship between gastrointestinal microbiota, muscle metabolites and meat quality. In this case, *p-*values *<0.05* and |r| values >0.05 were indicative of significant correlation between the parameters. Figdraw eventually allowed the mapping of the potential mechanisms that link gastrointestinal microbiota, muscle metabolites and meat quality.

## Results

### Carcass quality

The effects of adding RPSAA on the carcass quality of Tibetan sheep are shown in Table 1. Overall, there were no significant differences between the BSL, BH, CC, RFT and EMA of the three groups (*p > 0.05*). However, the BFT of the AP-2 and AP-3 groups was significantly higher than that of AP-1 (*p < 0.05*), with the AFT of AP-2 being also significantly higher than that of AP-1 (*p < 0.05*), and CW of AP-2 is significantly higher than that of AP-3 (*p < 0.05*). These results suggest that adding RPSAA can improve the carcass quality of Tibetan sheep.

**Table 1:** Effects of adding RPSAA on the carcass quality of Tibetan sheep.

Items	AP-1	AP-2	AP-3
Carcass weight (kg)	17.60 ± 0.20^a^	19.60 ± 1.11^a^	17.07 ± 0.92^b^
Body slope length (cm)	62.00 ± 3.00	62.33 ± 0.58	63.00 ± 1.00
Body height (cm)	64.00 ± 2.00	63.33 ± 2.89	73.67 ± 9.07
Chest circumference (cm)	78.00 ± 4.36	83.67 ± 2.08	79.33 ± 2.31
Rib fat thickness (cm)	2.19 ± 0.47	2.31 ± 0.14	1.75 ± 0.15
Back fat thickness (cm)	1.66 ± 0.25^b^	2.30 ± 0.09^a^	2.21 ± 0.17^a^
Abdominal fat thickness (cm)	2.11 ± 0.12^b^	2.50 ± 0.23^a^	2.30 ± 0.15^ab^
Eye muscle area (cm^2^)	17.83 ± 3.10	21.13 ± 4.25	20.62 ± 3.50

^a, b, c^ means that different letters on the same line indicate statistically significant differences (*p* < 0.05); the same letter means no significant difference (*p* > 0.05); the data in the table are all “mean ± SE”.

### Meat quality

#### Edible quality and nutritional composition of meat

As shown in Table 2, the addition of RPSAA had little effect on the pH_45min_, pH_24h_, redness (a^*^), yellowness (b^*^), thawing loss, cooked meat rate, water holding capacity and some texture parameters (elasticity, viscosity, cohesion, masticatory) of Tibetan sheep meat (*p > 0.05*). However, the brightness (L^*^) and cooking loss (CL) of the AP-2 group were significantly lower than those of the AP-1 group (*p < 0.05*). It is worth noting that there were significant differences in SF between the three groups (*p < 0.05*), with the order being as follows: AP-1 < AP-2 < AP-3. However, except for SF, other indicators were not significantly different between the AP-3 and the AP-1 groups. Meanwhile, the hardness of the AP-2 group was significantly higher than that of the other two groups (*p < 0.05*), while its adhesiveness was significantly higher than that of AP-1 (*p < 0.05*).

**Table 2:** Effects of adding RPSAA on the edible and nutritional quality of *longissimus lumborum* of Tibetan sheep.

Items	AP-1	AP-2	AP-3
pH_45min_	6.63 ± 0.29	6.70 ± 0.19	6.74 ± 0.34
pH_24h_	5.59 ± 0.51	5.94 ± 0.34	6.02 ± 0.28
L^*^	38.43 ± 1.94^a^	32.93 ± 2.77^b^	34.63 ± 2.13^ab^
a^*^	22.12 ± 2.75	19.43 ± 2.62	18.07 ± 2.66
b^*^	9.60 ± 2.92	9.43 ± 2.25	9.73 ± 1.14
Thawing loss (%)	6.58 ± 4.00	5.95 ± 1.36	5.95 ± 2.09
Cooking loss (%)	32.29 ± 1.51^a^	28.83 ± 2.22^b^	31.15 ± 0.85^ab^
Cooked meat percentage (%)	66.66 ± 0.45	66.81 ± 4.07	65.64 ± 2.65
Water holding capacity (%)	21.72 ± 1.40	27.84 ± 3.51	28.66 ± 7.37
Shear force (N)	64.07 ± 2.08^c^	75.32 ± 3.56^b^	96.87 ± 2.17^a^
Hardness (g)	1456.29 ± 228.48^b^	3179.60 ± 380.04^a^	1969.44 ± 476.16^b^
Elasticity (mm)	2.78 ± 0.31	2.40 ± 0.20	2.58 ± 0.16
Viscosity (mJ)	1.15 ± 0.53	0.59 ± 0.25	1.12 ± 0.37
Adhesion (g)	676.75 ± 142.08^b^	1233.17 ± 313.44^a^	978.04 ± 259.29^ab^
Cohesion	0.37 ± 0.06	0.41 ± 0.06	0.33 ± 0.05
Chewability (mJ)	19.58 ± 5.94	29.12 ± 4.96	23.55 ± 7.55
Moisture (%)	70.67 ± 1.20^b^	71.97 ± 0.63^a^	70.13 ± 1.02^ab^
Fat (%)	3.01 ± 1.10	2.68 ± 0.33	3.75 ± 1.31
Protein (%)	21.60 ± 1.98	21.19 ± 1.13	20.37 ± 0.28

^a, b, c^ means that different letters on the same line indicate statistically significant differences (*p* < 0.05); the same letter means no significant difference (*p* > 0.05); the data in the table are all “mean ± SE”.

Water, crude protein and crude fat are routinely present in muscles. As shown in Table 2, the addition of RPSAA did not significantly affect the fat and protein content of LL (*p > 0.05*), although in the case of the water content, that of AP-2 was significantly higher than that of AP-1 (*p < 0.05*). The above results suggest that adding RPSAA can increase the WHC, texture and SF of the LL muscle of Tibetan sheep.

#### AAs and FAs composition

The addition of RPAA had little effect on the amount of AAs and FAs in the LL of Tibetan sheep (Supplementary Tables S3, S4). As shown in Table 3, the TAAs, EAAs, NEAAs, SFAs, MUFAs and PUFAs content of the three groups did not differ significantly (*p > 0.05*), with the same results holding true for FAAs, SAAs and BAAs. However, compared with the other groups, the creatinine content of AP-1 and the aminoadipic acid content of AP-2 were significantly higher (*p < 0.05*). Furthermore, the C20:3 N6 content of AP-1 and AP-3 was significantly higher than that of AP-2 (*p < 0.05*), while the latter’s C20:5 N3, C22:5 N3, C22:6 N3 and ω-3 content was significantly lower than that of AP-1 (*p < 0.05*). Compared with AP-1, AP-2 also showed a decreasing trend in the amount of various FAs, while AP-3 was not significantly different in terms of its FA content, except in the case of C20:5 N3 which was significantly lower (*p < 0.05*).

**Table 3:** Effects of adding RPSAA on amino acids and fatty acids in the *longissimus lumborum* of Tibetan sheep.

Items	AP-1	AP-2	AP-3
Creatinine	0.13 ± 0.00^a^	0.11 ± 0.00^b^	0.11 ± 0.0^b^
aminoadipic acid	0.02 ± 0.00^b^	0.05 ± 0.01^a^	0.03 ± 0.00^b^
FAAs	3.55 ± 0.40	3.44 ± 0.37	3.12 ± 0.19
SAAs	8.81 ± 0.82	8.99 ± 0.57	8.79 ± 0.29
BAAs	3.63 ± 0.45	3.73 ± 0.13	3.68 ± 0.21
EAAs	2.41 ± 0.40	2.60 ± 0.17	2.37 ± 0.09
NEAAs	10.43 ± 0.79	10.32 ± 0.67	10.30 ± 0.24
TAAs	38.16 ± 4.58	40.85 ± 2.31	38.40 ± 1.87
C20:3 N6	2.22 ± 0.09^a^	1.56 ± 0.05^b^	1.93 ± 0.12^a^
C20:5 N3	3.48 ± 0.44^a^	2.20 ± 0.18^b^	2.30 ± 0.06^b^
C22:5 N3	5.53 ± 0.33^a^	4.05 ± 0.51^b^	5.15 ± 0.27^ab^
C22:6 N3	1.07 ± 0.03^a^	0.73 ± 0.15^b^	0.94 ± 0.05^ab^
SFAs	571.99 ± 202.08	322.89 ± 35.16	550.00 ± 142.29
MUFAs	731.82 ± 251.57	449.77 ± 71.79	706.76 ± 141.14
PUFAs	110.44 ± 15.14	70.94 ± 1.77	100.35 ± 15.25
n-3 PUFAs	16.71 ± 2.29^a^	9.88 ± 0.96^b^	14.14 ± 2.10^ab^
n-6 PUFAs	93.73 ± 13.01	61.06 ± 1.60	86.21 ± 13.21

^a, b, c^ means that different letters on the same line indicate statistically significant differences (*p* < 0.05); the same letter means no significant difference (*p* > 0.05). FAAs: Flavor amino acids; SAAs: Sweet amino acids; BAAs: bitter amino acids; EAAs: essential amino acids; NEAAs: nonessential amino acids; TAAs: total amino acids; SFAs: saturated fatty acids; MUFAs: monounsaturated fatty acids; PUFA: polyunsaturated fatty acids; complete amino acids data are shown in Supplementary Table S1, and fatty acids data are in Supplementary Table S2; the data in the table are all “mean ± SE”.

### Metabolomics analysis of the muscle

**Figure 1:**
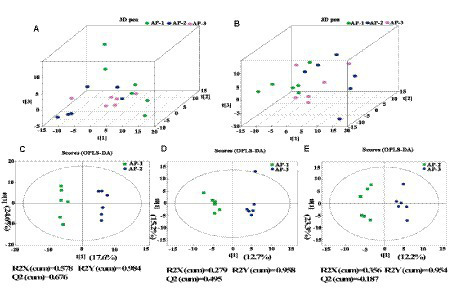
3D PCA scores of three samples in positive **(A)** and negative **(B)** ion detection modes; **(C)** (AP-1 and AP-2), **(D)** (AP-1 and AP-3), and **(E)** (AP-2 and AP-3) are the OPLS-DA score maps for pairwise comparison of three groups of samples under positive ion detection mode.

To further understand how RPMET and RPCYS supplementation influenced the quality of LL of the Tibetan sheep, non-targeted metabolites were detected with UHPLC-Q-TOF MS in the positive and negative ion modes, with the differences between the three groups shown by the three-dimensional PCA scores in Figures 1A,B. Moreover, to better investigate the group differences, orthogonal partial least squares discriminant analysis (OPLS-DA) was used to evaluate the metabolites in the meat samples. R2Y represents the cumulative variance value of the model, and the larger the value, the stronger the explanatory power of the model. Q2 represents the proportion of data variance predicted by the current model. Generally, if Q2 is greater than 0.5, it indicates that the model is stable and reliable. If 0.3 < Q2 < 0.5, it indicates good model stability. If Q2 < 0.3, it indicates low model reliability. As shown in Supplementary Table S5, R2Y in AP-1 VS AP-2 and AP-1 VS AP-3 is close to 1 and Q2 > 0.3, indicating that these two models have strong explanatory power and good stability (Kang et al., 2022). Figures 1C,D show obvious intra group aggregation and inter group dispersion. However, Q2 < 0.3 in the AP-2 vs. AP-3 model indicates that the reliability of the model is relatively low. Then, through 200 permutation and model overfitting tests (Supplementary Figure S2), it was found that the Q2 intercept regression line in the AP-1 vs. AP-2 and AP-1 vs. AP-3 models was less than 0, indicating that there was no overfitting phenomenon in the model. Therefore, the findings further confirm that the addition of RPMET and RPCYS has a significant impact on muscle metabolism in the LL of Tibetan sheep.

**Figure 2:**
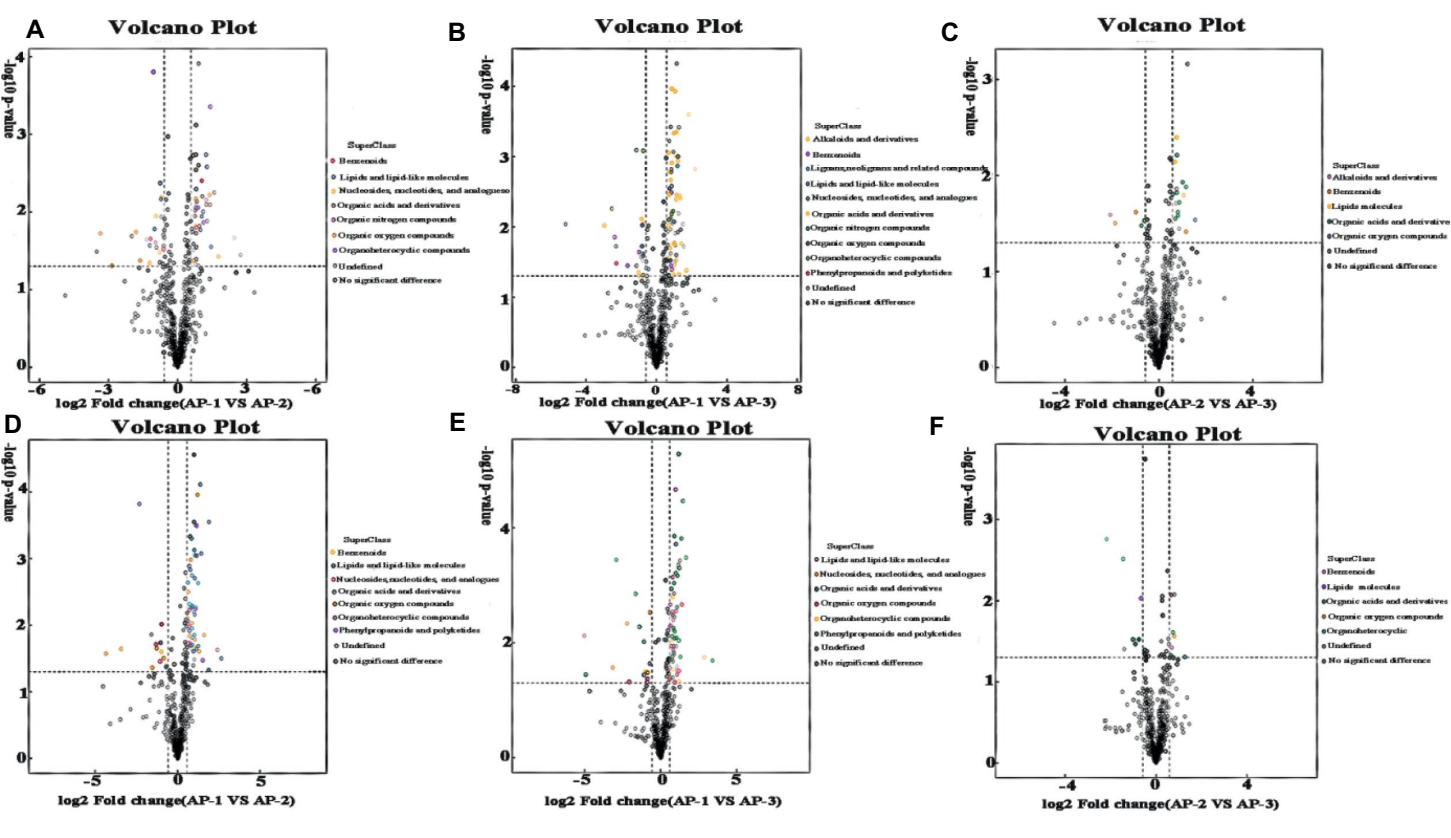
Volcanic maps of muscle metabolites in Tibetan sheep fed with RPSAA, with (**(A–C)**) showing the volcanic maps of AP-1 VS AP-2, AP-1 VS AP-3, and AP-2 VS AP-3 in positive ion mode, respectively; **(D–F)** are volcanic maps of AP-1 VS AP-2, AP-1 VS AP-3, and AP-2 VS AP-3 in negative ion mode, respectively.

**Figure 3:**
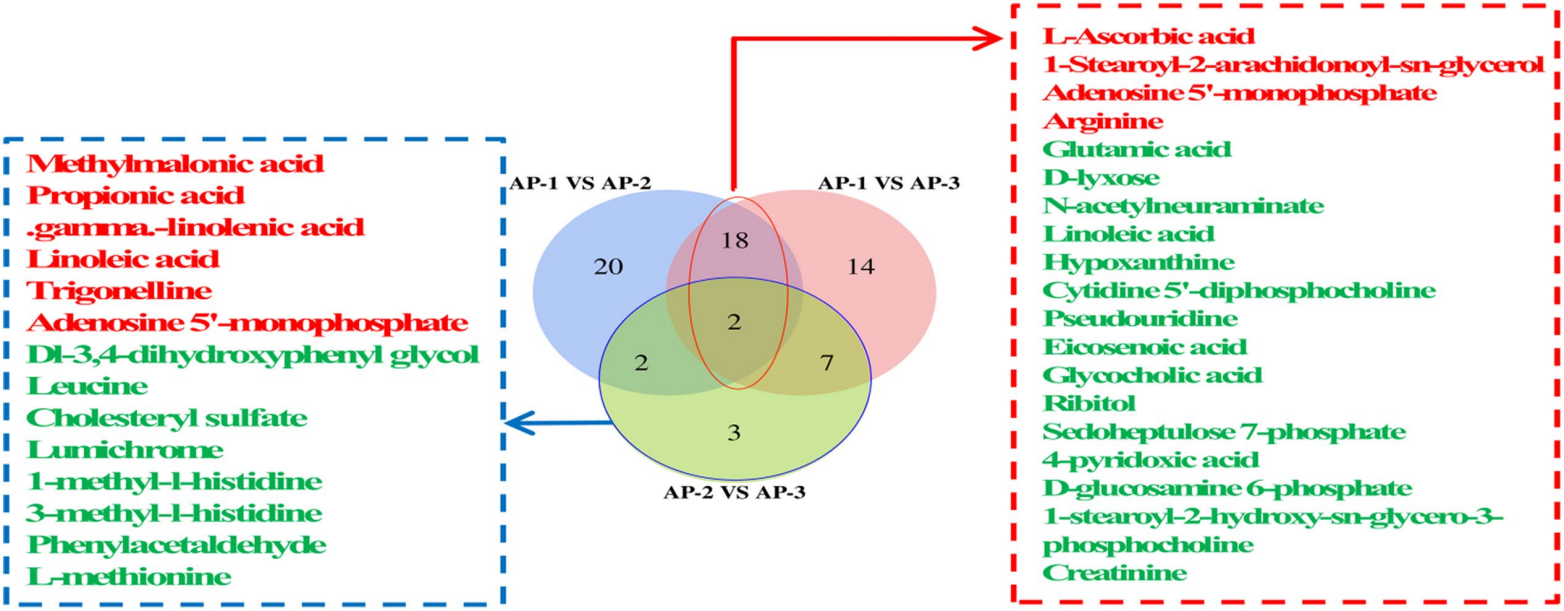
The Venn diagram shows a comparison of DMs involved in the KEGG pathway in the *longissimus lumborum* of three groups of Tibetan sheep; The red font indicates an increase in DMs, while the green font indicates a decrease in DMs.

Differential metabolites (DMs) between the three groups of samples at the super class level were observed on a volcanic map (Figure 2). In the positive ion mode, compared with AP-1, benzenoids, organic acids and derivatives as well as organic oxygen compounds (Figure 2A) were upregulated in AP-2, while lipids, lipid-like molecules and organic heterocyclic compounds were downregulated. On the other hand, alkaloids and derivatives, lipids and lipid-like molecules, nucleosides as well as nucleotides and analogues (Figure 2B) were upregulated in AP-3, while organic acids and derivatives were downregulated. When comparing AP-2 and AP-3, alkaloids and derivative, nucleosides, nucleotides and analytes were upregulated in the latter group (Figure 2C), while benzenoids, organic acids and derivatives as well as organic nitrogen compounds were downregulated. As far as the negative ion mode was concerned, compared with AP-1, organic oxygen compounds and other substances are upregulated in AP-2, while organic acids and derivatives, lipids and lipid-like molecules are downregulated (Figure 2D). In the meantime, lipids and lipid-like molecules, nucleosides, nucleotides as well as analogues were upregulated in AP-3, while organoheteric compounds, phenoylpropanoids, polyketides and organic oxygen compounds were downregulated (Figure 2E). And lastly when comparing AP-2 and AP-3, lipid-like molecules, organic acids, and derivatives were upregulated in AP-3, while benzenoids were downregulated (Figure 2F). In order to further understand the changes in DMs for the three groups of samples, specific metabolites from the three groups were compared in pairs and identified in the positive and negative ion modes based on a VIP of >1 and a *p-*value of *<0.05* as thresholds. A total of 163 DMs were found to be present in the three groups, of which 66 were involved in the KEGG pathway, as shown in Figure 3. The DM in the red box is common to the AP-1 and RPSAA groups (AP-2 and AP-3) after comparison, while the DM in the blue box is a comparison between AP-2 and AP-3 with added SAA. Compared with AP-1, 20 substances underwent changes in both AP-2 and AP-3. These included the upregulated L-ascorbic acid, adenosine 5′-monophosphate (AMP) and arginine as well as the downregulated glutamic acid, D-lyxose, hypoxantine, ribitol and sedoheptulose 7-phosphate, just to name a few. In addition, compared with AP-2, propionic acid, gamma-linolenic acid, linoleic acid and trigonelline were upregulated in AP-3, while leucine and L-methionine were downregulated. Overall, the changes in DMs in the three groups of samples influenced the upregulation or downregulation of metabolic pathways.

**Figure 4:**
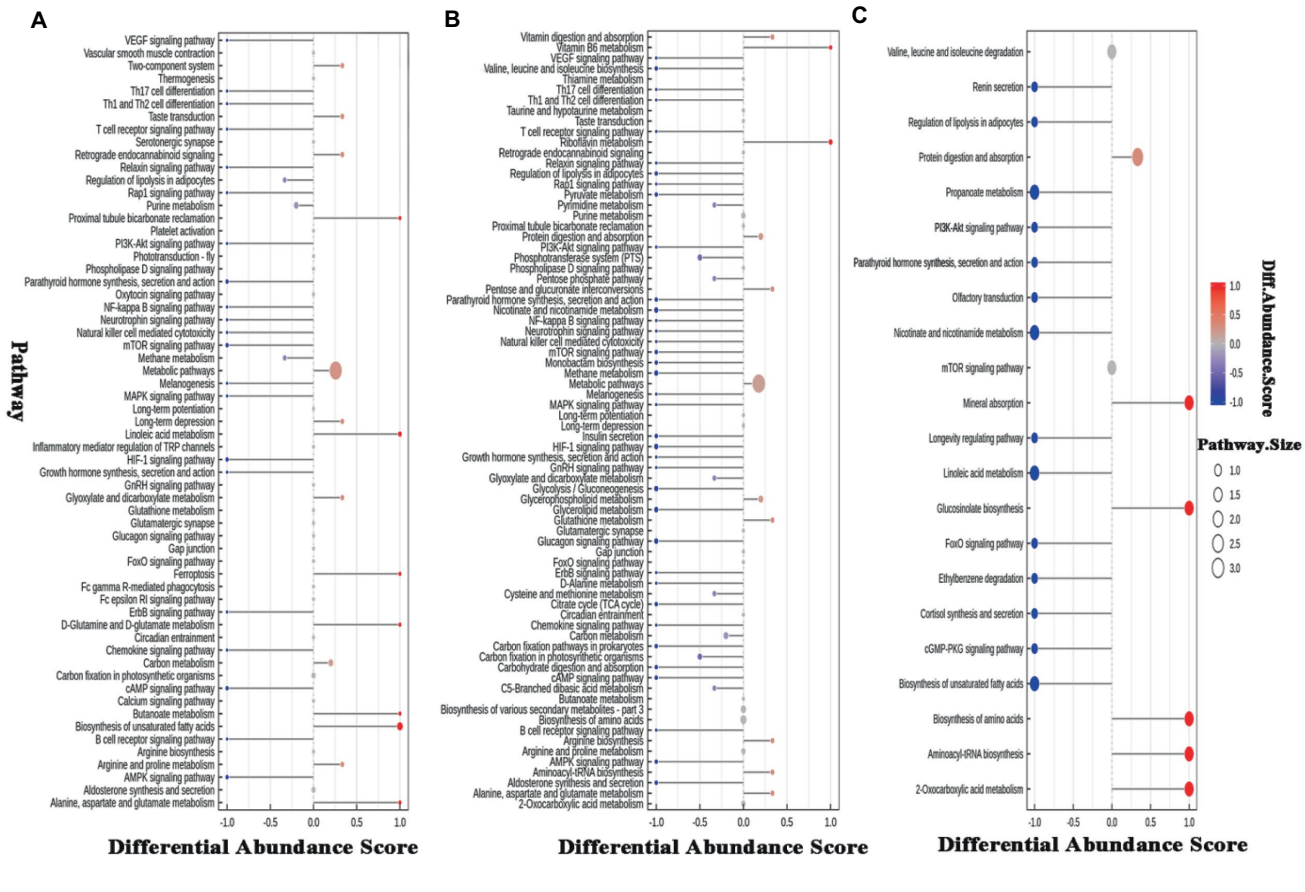
DA score map of differential metabolic pathways in the *Longissimus lumborum* of Tibetan sheep (**A**: AP-1 vs. AP-2, **B**: AP-1 vs. AP-3, **C**: AP-2 vs. AP-3). DA score is the overall total change of all metabolites in the metabolic pathway. In the figure, 1 indicates an upward trend in the expression of all identified metabolites in the pathway, while −1 indicates a downward trend in the expression of all identified metabolites in the pathway.

To further understand the impact of adding RPSAA on the metabolic pathways of Tibetan sheep, the three groups were analyzed using Differential Abundance Scores (DA score). As shown in Figure 4, compared with AP-1, 39 metabolic pathways were upregulated and 2 were downregulated in AP-3, while for AP-2, 21 were upregulated and 7 were downregulated. Of these, 21 were upregulated in both groups and included metabolic pathways such as parathyroid homone synthesis, secret and action, growth homone synthesis, secret and action, melanogenesis as well as eight important signaling pathways (e.g., mTOR signaling pathway, HIF-1 signaling pathway, etc.). Meanwhile, compared with AP-1, the upregulated metabolic pathways in AP-3 included glycolysis/gluconeogenesis, citrate cycle (TCA cycle), pyruvate metabolism, valine, leucine and isoleucine biosynthesis and glycerolide metabolism, just to name a few, with sedoheptulose 7-phosphate, pyruvate, phosphoenolpyruvate, 2-phosphoglycerate, propionic acid, trigonelline, glycerophosphate (2), glycerol 3-phosphate and citronic acid being some of the upregulated metabolites within these metabolic pathways (Table 4). On the other hand, the downregulated pathways in AP-3 included riboflavin and vitamin B6 metabolism, with the corresponding downregulated metabolites including flavone mononucleotide, lumichrome, ribitol, 4-pyridoxic acid and pyridoxal phosphate (Table 4). As far as AP-2 was concerned, the biosynthesis of unsaturated fatty acids, linoleic acid metabolism, alanine, aspartate and glutamate metabolism as well as butanoate metabolism were some of the pathways that were downregulated compared with AP-1, while the downregulated metabolites included linoleic acid, arachidonic acid (peroxide free), palmitic acid, eicosenoic acid, gamma-linolenic acid, nervonic acid, glutamic acid and D-pyroglutamic acid, amongst others (Table 4).

**Table 4:** Changes in differential metabolites in key metabolic pathways compared among three groups of samples (absolute difference abundance score of all metabolic pathways ≥0.5).

Metabolic pathways	Metabolites
**Upregulation in the AP-2 group**	**AP-1 VS AP-2**
mTOR signaling pathway	Adenosine 5′-monophosphate, Arginine
HIF-1 signaling pathway	Stearoyl-2-arachidonoyl-sn-glycerol L-Ascorbic acid
AMPK signaling pathway	Adenosine 5′-monophosphate Fructose 1,6-diphosphate
PI3K-Akt signaling pathway	Adenosine 5′-monophosphate
**Downregulation in the AP-2 group**	
Biosynthesis of unsaturated fatty acids	11,14,17-eicosatrienoic acid, (z,z,z)-Linoleic acid, Arachidonic acid (peroxide free) Palmitic acid, Eicosenoic acid gamma-linolenic acid, Nervonic acid
Linoleic acid metabolism	Arachidonic acid (peroxide free) gamma-linolenic acid, Linoleic acid
Butanoate metabolism	Glutamic acid, Maleic acid
Alanine, aspartate and glutamate metabolism	Glutamic acid, D-glucosamine 6-phosphate
**Upregulation in the AP-3 group**	**AP-1 VS AP-3**
HIF-1 signaling pathway	Pyruvate, L-Ascorbic acid 1-Stearoyl-2-arachidonoyl-sn-glycerol
mTOR signaling pathway	Adenosine 5′-monophosphate, Arginine
AMPK signaling pathway	Pyruvate, Adenosine 5′-monophosphate
PI3K-Akt signaling pathway	Adenosine 5′-monophosphate
Glycolysis / Gluconeogenesis	Pyruvate, 2-phosphoglycerate Phosphoenolpyruvate
Citrate cycle (TCA cycle)	Pyruvate, Phosphoenolpyruvate
Pyruvate metabolism	Pyruvate, Phosphoenolpyruvate
Valine, leucine and isoleucine biosynthesis	Pyruvate, Citraconic acid
Glycerolipid metabolism	Glycerophosphate (2), Glycerol 3-phosphate 2-phosphoglycerate
Nicotinate and nicotinamide metabolism	Propionic acid, Pyruvate, Trigonelline
**Downregulation in the AP-3group**	
Riboflavin metabolism	Flavine mononucleotide, Lumichrome, Ribitol
Vitamin B6 metabolism	4-pyridoxic acid, Pyridoxal phosphate
**Upregulation in the AP-3 group**	**AP-2 VS AP-3**
Linoleic acid metabolism	gamma-linolenic acid, Linoleic acid
Biosynthesis of unsaturated fatty acids	gamma-linolenic acid, Linoleic acid
Nicotinate and nicotinamide metabolism	Propionic acid, Trigonelline
FoxO signaling pathway	Adenosine 5′-monophosphate
Regulation of lipolysis in adipocytes	Adenosine 5′-monophosphate
**Downregulation in the AP-3group**	
Biosynthesis of amino acids	Leucine, L-methionine
2-Oxocarboxylic acid metabolism	Leucine, L-methionine

As shown in Figure 4, in comparison with AP-2, AP-3 had 14 upregulated and 5 downregulated metabolic pathways. More specifically, linoleic acid metabolism, propanoate metabolism, nicotinate and nicotinamide metabolism, biosynthesis of unsaturated fatty acids and their related signaling pathways (PI3K-Akt signaling pathway, FoxO signaling pathway, cGMP-PKG signaling pathway) were upregulated, while the biosynthesis of amino acids and 2-Oxocarboxylic acid metabolism were some of those that were downregulated. Within these key metabolic pathways, gamma-linolenic acid, linoleic acid, methylmalonic acid, propionic acid, trigonelline and AMP were some of the upregulated metabolites, while leucine and L-methionine were among the downregulated ones (Table 4).

### Analysis of the composition of gastrointestinal microbiota

#### Analysis of gastrointestinal fermentation parameters

Results, shown in Table 5, indicated the absence of significant differences between the pH, ammoniacal nitrogen and rumen fluid’s SCFAs of the three groups (*p > 0.05*). However, it was noted that the ammoniacal nitrogen content of the AP-1 group without RPAA addition was higher than that of AP-2 and AP-3 in which RPSAA was added. In addition, the SCFAs content of AP-2 and AP-3 also showed an upward trend compared with AP-1. The parameters of jejunal fermentation were significantly different between the three groups (Table 5). However, the ammoniacal nitrogen content in AP-1 was significantly higher than that of AP-2 and AP-3, while the valeric acid content increased significantly in AP-3 (*p < 0.05*). There were also significant differences in butyric acid content between the three groups, with the highest amount being in the AP-3 group, followed by AP-2 (*p < 0.05*).

**Table 5:** Effects of adding PRSAA on rumen and jejunum fermentation characteristics of Tibetan sheep.

Items	AP-1	AP-2	AP-3
Rumen			
pH	6.34 ± 0.18	6.58 ± 0.22	6.25 ± 0.17
NH_3_-N (mg/100 mL)	27.33 ± 3.49	20.28 ± 4.06	21.81 ± 2.06
Acetic acid (mmol/L)	287.50 ± 45.28	405.30 ± 162.39	466.04 ± 150.51
Propionic acid (mmol/L)	118.15 ± 27.14	196.93 ± 57.58	173.92 ± 23.19
Isobutyric acid (mmol/L)	4.13 ± 0.54	7.35 ± 1.83	9.01 ± 2.85
Butyric acid (mmol/L)	18.15 ± 1.72	28.90 ± 6.67	38.12 ± 11.66
Isovaleric acid (mmol/L)	6.41 ± 1.18	10.55 ± 2.60	15.95 ± 5.76
Valeric acid (mmol/L)	5.05 ± 0.84	7.11 ± 2.24	8.83 ± 2.61
TSCFAs (mmol/L)	438.84 ± 90.98	656.14 ± 284.09	711.87 ± 176.13
A/P (mmol/L)	2.64 ± 0.76	2.12 ± 0.22	2.80 ± 1.26
Jejunum			
NH_3_-N (mg/100 mL)	5.71 ± 1.06^a^	2.27 ± 1.15^b^	2.14 ± 0.79^b^
Acetic acid (mmol/L)	5.65 ± 1.33	7.44 ± 2.99	5.89 ± 0.60
Propionic acid (mmol/L)	1.25 ± 0.18	1.60 ± 0.20	1.51 ± 0.16
Isobutyric acid (mmol/L)	0.21 ± 0.04	0.21 ± 0.01	0.21 ± 0.02
Butyric acid (mmol/L)	0.27 ± 0.01^c^	0.57 ± 0.06^a^	0.42 ± 0.01^b^
Isovaleric acid (mmol/L)	1.06 ± 0.49	1.20 ± 0.46	1.32 ± 0.19
Valeric acid (mmol/L)	0.32 ± 0.12^b^	0.69 ± 0.21^a^	0.39 ± 0.05^b^
TSCFAs (mmol/L)	8.76 ± 1.65	11.75 ± 3.66	9.76 ± 0.83
A/P (mmol/L)	4.49 ± 0.39	4.82 ± 1.46	3.92 ± 0.37

^a, b, c^ means that different letters on the same line indicate statistically significant differences (*p* < 0.05); the same letter means no significant difference (*p* > 0.05); TSCFAs represents the total content of short chain fatty acids; A represents acetic acid, *p* represents propionic acid; the data in the table are all “mean ± SE”.

#### Analysis of rumen microbiota composition

**Figure 5:**
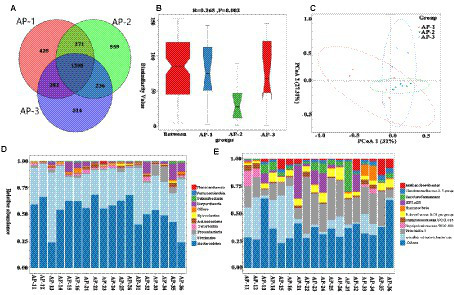
OTUs Vene plots of three groups of rumen microorganisms **(A)**. Analysis of variance **(B)** and PCoA plot **(C)** of the overall sample of rumen microorganisms. The relative abundance of bacterial community proportions at the phylum **(D)** and genus **(E)** levels in three sets of samples.

As shown in Figure 5A, a total of 3,684 OTUs were detected in the rumen, of which 425, 559 and 514 were specific to AP-1, AP-2 and AP-3, respectively. Comparing the α diversity index (Supplementary Table S6) of the three groups showed that the Shannon and Simpson values of AP-2 were significantly higher than those of AP-1 (*p < 0.05*), hence indicating that the flora diversity of AP-2 was significantly higher. Anosim (Figure 5B) and PCoA (Figure 5C) analyses subsequently showed significant differences and good dispersion between the bacterial communities of the three groups. At the phylum level, the main rumen microbiota were *Firmicutes* and *Bacteroidetes* (Figure 5D), while at the genus level, the main groups were *uncultured rumen bacterium*, *Prevotella 1*, and *Rikenellaceae RC9 gut group* (Figure 5E). Table 6 shows the main differences in the rumen microbiota at the phylum and genus level for the three groups. Firstly, the abundance of *Bacteroidetes* in AP-2 was significantly higher than that of AP-3 (*p* < 0.05), while the proportion of *Proteobacteria* in AP-3 was significantly higher than for the other two groups. At the genus level, the abundance of *Prevotella 1* and *Rikenellaceae RC9 gut groups* in AP-2 was significantly higher than that in AP-1 (*p < 0.05*), while the abundance of the *Lachnospiraceae NK4A136* and *Lachnospiraceae ND3007* groups was significantly higher than that of AP-1 and AP-3 (*p < 0.05*). However, the abundance of the *Christensenellaceae R-7* group, *uncultured*, *Lachnospiraceae NK3A20* group, *Prevotellaceae UCG-003*, *Acetomaculum* and *Rumnococcaceae UCG-011* in AP-3 was significantly higher than for the other two groups (*p < 0.05*). Meanwhile, *Desulfovibrio* and U29-B03 were significantly reduced in AP-2 and AP-3, respectively (*p < 0.05*).

**Table 6:** Differences in phylum and genus levels of bacteria in three groups of rumen (accounting for the top 15 relative abundances).

Items	AP-1	AP-2	AP-3
Phylum level (%)			
*Bacteroidetes*	54.85 ± 15.66^ab^	61.53 ± 5.83^a^	43.49 ± 10.65^b^
*Firmicutes*	35.92 ± 16.06	29.52 ± 5.28	38.58 ± 13.59
*Tenericutes*	1.18 ± 0.49	1.65 ± 1.12	1.21 ± 1.51
*Proteobacteria*	1.84 ± 0.79^b^	1.52 ± 1.25^b^	6.01 ± 5.22^a^
*Actinobacteria*	0.97 ± 0.38	1.62 ± 1.29	1.79 ± 1.16
Genus level (%)			
*Prevotella 1*	12.88 ± 2.33^b^	31.08 ± 5.66^a^	23.07 ± 3.41^ab^
*uncultured rumen bacterium*	31.23 ± 6.65^a^	6.50 ± 2.55^b^	5.98 ± 2.69^b^
*Rikenellaceae RC9 gut group*	4.36 ± 1.01^b^	7.59 ± 0.96^a^	4.92 ± 0.79^ab^
*Christensenellaceae R-7 group*	1.76 ± 0.42^b^	1.71 ± 0.38^b^	3.50 ± 0.56^a^
*Uncultured*	0.64 ± 0.11^b^	0.59 ± 0.03^b^	1.00 ± 0.16^a^
*Lachnospiraceae NK3A20 group*	0.34 ± 0.08^b^	0.37 ± 0.03^b^	0.93 ± 0.20^a^
*Prevotellaceae UCG-003*	0.34 ± 0.07^b^	0.39 ± 0.03^b^	0.66 ± 0.08^a^
*Erysipelotrichaceae UCG-004*	0.11 ± 0.02	0.16 ± 0.04	0.33 ± 0.17
*Acetitomaculum*	0.11 ± 0.03^b^	0.12 ± 0.01^b^	0.32 ± 0.07^a^
*[Eubacterium] ruminantium group*	0.61 ± 0.10^ab^	0.98 ± 0.19^a^	0.31 ± 0.08^b^
*Ruminococcaceae UCG-011*	0.03 ± 0.01^b^	0.06 ± 0.02^b^	0.27 ± 0.12^a^
*Desulfovibrio*	0.12 ± 0.03^ab^	0.07 ± 0.01^b^	0.19 ± 0.05^a^
*Lachnospiraceae NK4A136 group*	0.08 ± 0.02^b^	0.30 ± 0.09^a^	0.11 ± 0.04^b^
*Lachnospiraceae ND3007 group*	0.09 ± 0.01^b^	0.38 ± 0.15^a^	0.11 ± 0.02^b^
*U29-B03*	0.20 ± 0.03^ab^	0.26 ± 0.06^a^	0.10 ± 0.03^b^

^a, b, c^ means that different letters on the same line indicate statistically significant differences (*p* < 0.05); the same letter means no significant difference (*p* > 0.05); the data in the table are all “mean ± SE”.

#### Analysis of jejunal microbiota composition

**Figure 6:**
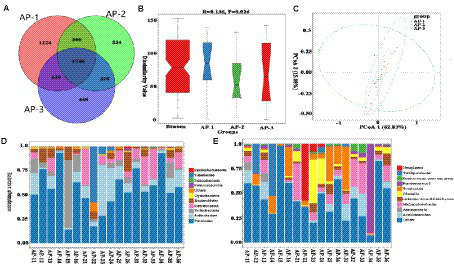
OTUs Vene plots of three groups of jejunal microbiota **(A)**. Analysis of variance **(B)** and PCoA plot **(C)** of the overall sample of jejunal microbiota. The relative abundance of bacterial community proportions at the phylum **(D)** and genus **(E)** levels in three sets of samples.

As shown in Figure 6A, a total of 5,039 OTUs were detected in the jejunum, of which 1,224, 534 and 448 were specific to AP-1, AP-2 and AP-3, respectively. The α diversity index (Supplementary Table S7) of the three groups was not significantly different, hence indicating the absence of significant changes in the diversity and abundance of the jejunum flora of the three groups. Anosim (Figure 6B) and PCoA (Figure 6C) analyses further highlighted significant differences between the three bacterial groups. Unlike the rumen, the dominant phyla in the jejunum were *Firmicutes* (Figure 6D), with Table 7 showing the main differences in jejunal microbiota at the phylum and genus level between the three groups. At the phylum level, there was no significant differences in the relative abundance of the top five phyla. However, at the genus level (Figure 6E), the abundance of *Olsenella*, *Eubacterium nodatum group* and *Mogibacterium* in AP-2 were significantly higher than that of AP-1 (*p < 0.05*). Additionally, the proportion of *[Eubacterium] Brachy group* and *CAG-352* in AP-2 were significantly higher than for the other two groups (*p < 0.05*). Finally, the abundance of *Turicibacter* and *Atopobium* was highest in AP-1 and AP-3, respectively (*p < 0.05*).

**Table 7:** Differences in phylum and genus levels among three groups of jejunal bacteria (accounting for the top 7 relative abundances).

Items	AP-1	AP-2	AP-3
Phylum level (%)			
*Firmicutes*	58.85 ± 26.91	42.28 ± 17.32	65.60 ± 16.31
*Proteobacteria*	18.91 ± 25.80	3.51 ± 2.53	4.28 ± 5.87
*Actinobacteria*	8.64 ± 8.40	21.43 ± 18.46	9.11 ± 6.08
*Bacteroidetes*	8.02 ± 5.34	6.12 ± 7.69	3.74 ± 3.53
*Euryarchaeota*	3.79 ± 5.73	11.28 ± 13.58	15.35 ± 12.26
Genus level (%)			
*Olsenella*	1.25 ± 0.46^b^	14.38 ± 7.09^a^	2.84 ± 0.87^ab^
*[Eubacterium] nodatum group*	0.15 ± 0.04^b^	0.43 ± 0.12^a^	0.34 ± 0.09^ab^
*Mogibacterium*	0.11 ± 0.04^b^	0.38 ± 0.10^a^	0.33 ± 0.10^ab^
*[Eubacterium] brachy group*	0.08 ± 0.02^b^	0.20 ± 0.06^a^	0.03 ± 0.01^b^
*CAG-352*	0.02 ± 0.01^b^	0.19 ± 0.08^a^	0.05 ± 0.01^b^
*Turicibacter*	0.72 ± 0.32^a^	0.15 ± 0.07^b^	0.04 ± 0.02^b^
*Atopobium*	0.16 ± 0.07^b^	0.13 ± 0.04^b^	0.60 ± 0.21^a^

^a, b, c^ means that different letters on the same line indicate statistically significant differences (*p* < 0.05); the same letter means no significant difference (*p* > 0.05); the data in the table are all “mean ± SE”.

### Correlation analysis

**Figure 7:**
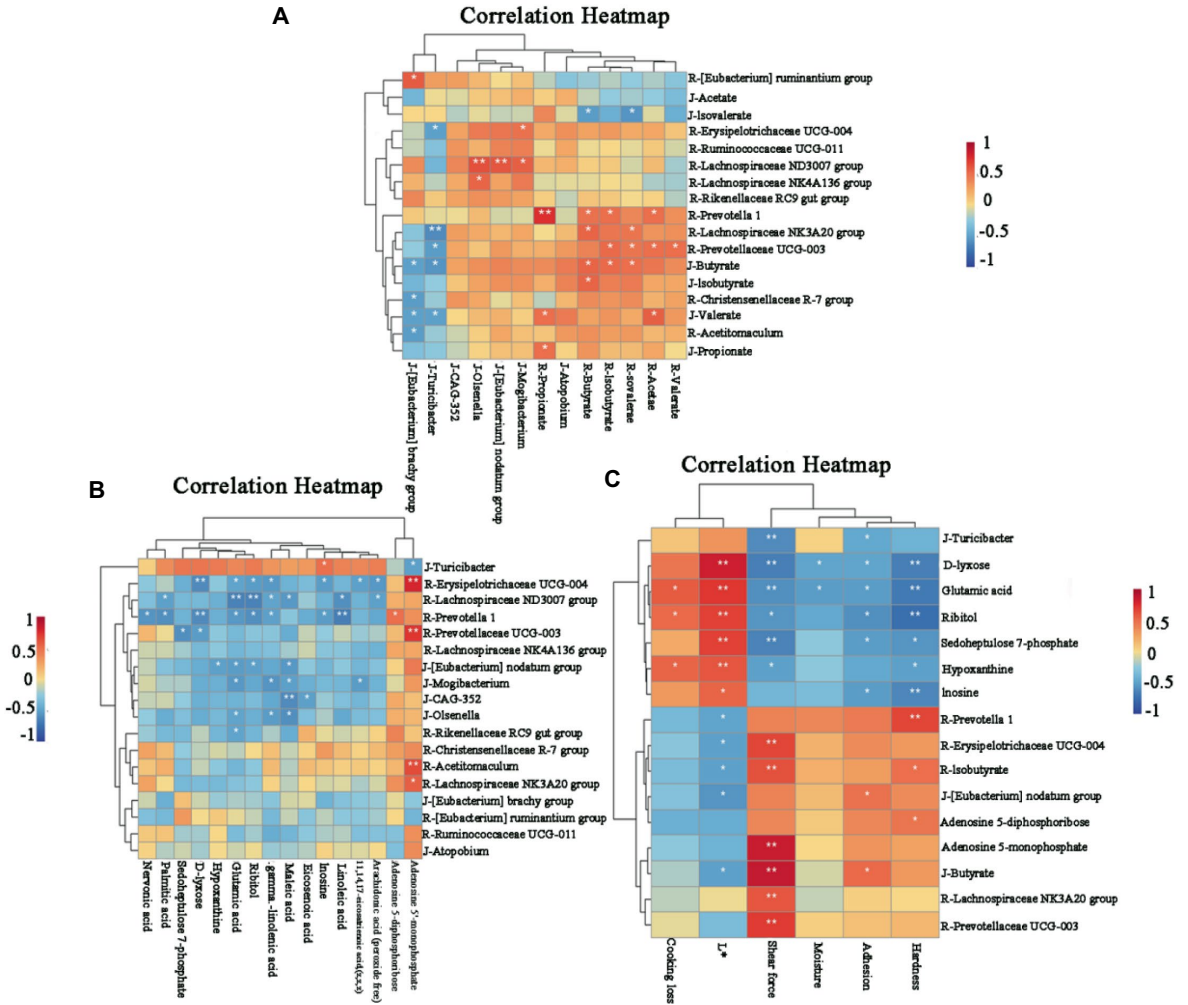
Cluster heatmap of correlation analysis between rumen and jejunum microbiota and SCFAs **(A)**. Cluster heatmap of correlation analysis between rumen and jejunal microbiota and metabolites of the *Longissimus lumborum* in Tibetan sheep **(B)**. Cluster heatmap of correlation analysis between rumen and jejunal microbiota and metabolites of the *Longissimus lumborum* in Tibetan sheep **(C)**. The microbiota and SCFAs with R before them represent the rumen, while the J before them represents the jejunum.

Figure 7A shows the correlation analysis of rumen microbiota, SCFAs and jejunal microbiota. Acetic acid, butyric acid, isobutyric acid, valeric acid and isovaleric acid in the rumen were positively correlated with *Prevotella 1, Lachnospiraceae NK3A20 group* and *Prevotella UCG-003*. On the other hand, the levels of butyric acid and valeric acid in the jejunum were negatively correlated with the *[Eubacterium] Brachy group* and *Turicibacter*. At the same time, the *Lachnospiraceae NK3A20 group* and *Prevotellaceae UCG-003* in the rumen were negatively correlated with the *[Eubacterium] Brachy group* and *Turicibacter* in the jejunum, while the butyric acid and valeric acid in the jejunum were positively correlated with acetic acid, butyric acid, isobutyric acid and isovaleric acid in the rumen. Therefore, it was speculated that the *Lachnospiraceae NK3A20 group* and *Prevotellaceae UCG-003* in the rumen could have an impact on the *[Eubacterium] Brachy group* and *Turicibacter* in the jejunum, thereby influencing the production of SCFAs.

Figure 7B shows the correlation analysis between microorganisms and muscle metabolites in the rumen and jejunum. Firstly, it was found that the *Lachnospiraceae NK3A20* group was only positively correlated with AMP, while *Prevotellaceae UCG-003* had a positive correlation with AMP, D-lyxose, Sedoheptulose 7-phosphate. On the other hand, *Tubriciactor* was positively correlated with inosine but negatively correlated with AMP. *Prevotella 1, Erysipelotrichaceae UCG-004* and *Lachnospiraceae ND3007* groups in the rumen were also positively correlated with arachidonic acid (peroxide free), 11,14,17-eicosatrienoic acid (z, z, z)-gamma-linolenic acid, palmitic acid, glutamic acid, maleic acid. Finally, the *[Eubacterium] nodatum group* in the jejunum was negatively correlated with hypoxanthine, glutamic acid and ribitol, while a negative correlation was noted between *Mogibacterium,* 11,14,17-eicosatrienoic acid, (z, z, z)-gamma-linolenic acid and glutamic acid.

Results of correlation analyses between the apparent quality of meat, muscle metabolites as well as rumen and jejunal microbiota are shown in Figure 7C. It was noted that D-lyxose, glutamic acid, ribitol, sedoheptulose 7-phosphate, hypoxantine and inosine were positively correlated with L^*^, and negatively correlated with SF. While adenosine 5′-diphophosphoribose (ADP) and AMP were negatively correlated with L^*^, and positively correlated with SF. At the same time, *Prevotella 1* and *Erysipelotrichaceae UCG-004* in the rumen were positively correlated with L^*^, while isobutyric acid in the rumen and butyric acid in the jejunum were negatively correlated with L^*^, while SF was completely opposite.

## Discussion

MET and CYS are SAA, and there is a complex transformation relationship between them. MET is the precursor of CYS, which can transfer methyl through the intermediate metabolite S-adenosylmethionine to form S-adenosylhomocysteine, then it is hydrolyzed to homocysteine, and finally CYS is generated through sulfur transformation (Tang et al., 2010). In this context, research by Finkelstein (1990) suggested that CYS can replace more than half of the MET in the diet due to their turnover mechanism in the body. MET and CYS are precursors of glutathione, taurine and sub taurine, which have been proved to have strong antioxidant capacity (Wu et al., 2004; Kotzamanis et al., 2019). Good redox state can make animal meat show better quality characteristics in water holding capacity, color stability and anti lipid and protein oxidation (Estévez, 2015). Therefore, through the control of animal nutrition, MET and CYS can be reasonably used to improve meat quality. Adding amino acids to diets is beneficial for animal growth, production and reproduction. However, supplementing feeds with high levels of amino acids may also cause imbalance or antagonism, thereby inhibiting growth (Peng et al., 1973). Similarly, insufficient amounts of amino acids can reduce weight gain, feed efficiency and feed intake (Castro et al., 2020). This study set up three groups, namely the group without added RPSAA (AP-1 group), the group with added RPMET (AP-2 group), and the group with added RPMET and RPCYS (AP-3 group), in order to clarify the effects of RPSAA on rumen microbiota, meat metabolism, and meat quality in Tibetan sheep.

In this study, the proportion of RPSAA added in this study has no adverse effect on the carcass quality of Tibetan sheep. The color of meat is closely related to its freshness as it directly reflects the quality of meat and influences consumers’ acceptance (Ripoll and Panea, 2019). Meat that is popular among consumers generally exhibits a bright cherry red color (Sawyer et al., 2009). However, when the values of a^*^ and b^*^ are not significantly different, a significant decrease in L^*^ can result in a dark red state. In this context, research has found that the L^*^ of meat was related to its muscle fat content and WHC. For instance, Silva et al. found that a decrease in beef meat’s brightness was accompanied by a lower intramuscular fat (Silva et al., 2019). In this study, the L^*^ value of AP-2 decreased significantly, while its fat content was the lowest. As already pointed out, the amount and distribution of water in muscle tissues can also affect the color of meat. Muscles contract when they become stiff after death, and water, from the muscle fiber compartment, enters the intercellular space followed by the extracellular space (Honikel et al., 1986). An increase in muscle contraction therefore leads to an increase in the water leakage which subsequently affects the refractive index of the muscle surface and leads to an increase in L^*^. In this study, AP-2 also had the lowest cooking loss and the highest moisture content, and hence the lowest L^*^.

Linda et al. found that in color-stable lamb loin, a decrease in inosinemonphosphate (IMP) was accompanied by a significant increase in inosine concentration (Samuelsson et al., 2022). This is because after sheep are slaughtered, ATP in the meat forms IMP through ADP and AMP, and then further forms hypoxanthine through inosine (Yano et al., 1995). During the process of muscle conversion into meat, the initial concentrations of AMP and IMP in the meat are high, while the inosine and hypoxanthine content is low, resulting in poor tenderness of the meat. At later stages, ATP reserves are depleted and secondary energy consumption begins. This involves the decomposition of AMP and IMP into inosine and hypoxanthine, resulting in an increase in meat tenderness (Graham et al., 2012; Beldarrain et al., 2023). Interestingly, in the correlation analysis, there was a significant correlation between L^*^ and SF with purine metabolism and metabolites in its surrounding pathways, as well as with the microbiota and SCFAs in the rumen and jejunum (Figure 7C). So far, research has revealed that the gastrointestinal microbiota could regulate meat quality by producing SCFAs (Zhang et al., 2022a; Dou et al., 2023). Therefore, it was speculated that feeding Tibetan sheep with RPSAA could influence purine metabolism and its nearby pathways by altering the gastrointestinal microbiota and increasing the production of SCFAs, thereby reducing L^*^ and increasing SF.

In recent years, people have been paying attention to both the nutritional needs of animals as well as the tenderness of their meat. Tenderness is an important quality attribute that also affects consumers’ purchase of meat, and it is generally determined through a sensory evaluation or measurement of SF (Warner et al., 2021). In their study of meat tenderness, Haiqing et al. found that adding different concentrations of RPMET to the diet of Tan sheep did not significantly affect the SF (Li et al., 2020). In fact, the study by Zhiyuan et al. showed that low to medium concentrations of RPLYS and RPMET significantly reduced the SF of yak meat (Ma et al., 2021). On the other hand, in this study, the addition of RPSAA was found to significantly increase the SF of Tibetan sheep meat. The above findings suggest that the role of RPAA may vary according to the animal species, types of amino acids and feeding background. The tenderness of meat is also related to its final pH value (pHu), with values ranging from 5.8 to 6.19 corresponding to a higher meat SF compared with those which are ≤5.79 or ≥ 6.2. This could be attributed to the fact that, within this pH range, small heat shock proteins are not easily degraded. At the same time, cathepsin B and μ-Calpain have low activities which further increase the toughness of meat and delay its tenderness (Lomiwes et al., 2013). In this study, the pHu of the RPSAA group was between 5.8 and 6.19. This could explain why the SF of this group was greater compared with that of the ordinary amino acid group. However, it should be noted that the maximum SF of the LL samples did not exceed 11 kgF, with Bickerstaffe suggesting that this value was an acceptable upper limit for cooked meat tenderness (Bickerstaffe et al., 2001).

In recent years, the relationship between cell apoptosis and tenderness attracted much attention. In this context, the study by Cheng Chen et al. revealed that cell apoptosis, mediated by apoptosis inducing factor (AIF), could increase the tenderness of beef muscle (Chen et al., 2020). Similarly, Zhang et al. found that inducing higher cell apoptosis through diet could increase the tenderness of meat, while delaying the apoptotic process resulted in a tougher meat texture (Ma et al., 2020; Zhang et al., 2022). After animal slaughter, muscle cells enter a state of hypoxia during which they produce a hypoxic stress response that activates various signaling pathways. This, in turn, gradually leads to a change from oxidative respiration to anaerobic glycolysis to provide energy for cells (Wang et al., 2022). However, hypoxic stress also inevitably produces ROS and induces HIF-1α accumulation through the PI3K/AKT–mTOR signaling pathway (Tang and Zhao, 2020; Chen et al., 2022). HIF-1 is an important regulatory pathway for cell apoptosis, and it can regulate metabolic adaptation, metastasis and anti-apoptosis of hypoxic cells (Paik et al., 2017). It was found that an increase in HIF-1α expression was accompanied by an increase in sarcoplasmic calcium (Ca^2+^) during postmortem maturation. Ca^2+^, through calmodulin CaMKK β activating AMPK, promotes the transfer of GLUT1 to the cell membrane and the phosphorylation of transcription factors to induce glucose uptake and transport. As a result, this process maintains energy production in cells under hypoxic conditions while inhibiting cell apoptosis (Gao et al., 2019; Xin et al., 2023). Therefore, it can be inferred that the AMPK pathway can regulate the *post mortem* glycolysis process. In this study, the PI3K-Akt, mTOR, HIF-1 and AMPK signaling pathway as well as the metabolites in the RPSAA groups were upregulated compared with the AP-1 group. Hence, it was speculated that, after slaughter, the activation and upregulation of these pathways in the RPSAA group of Tibetan sheep could delay cell apoptosis and meat tenderization due to hypoxic stress. However, further research would be required to indeed ascertain the above speculation.

On the other hand, it was found that, in the RPCYS group, glycolysis/gluconeogenesis and the citrate cycle (TCA cycle) were upregulated, hence indicating that aerobic respiration and glycolysis were the two mechanisms through which energy was provided to muscle cells at this time. These findings also suggested that the muscle was in an early postmortem stage (Matarneh et al., 2018). Upregulation of the glycolysis pathway generally increases lactate concentration, thereby reducing pH and increasing meat hardness (Chen et al., 2019). However, in the RPCYS group, the meat texture was harder although the pH value was higher than for the other two groups. This could be due to the slower glycolysis rate that prolonged the zombie stage and slowed down the softening rate of the meat (Honikel, 2014). Therefore, this study suggests that the acceleration of glycolysis rate, followed by a rapid decrease in pH, can be beneficial for the hydrolysis of muscle fiber proteins as well as for improving the tenderness of meat (Barón et al., 2021).

**Figure 8:**
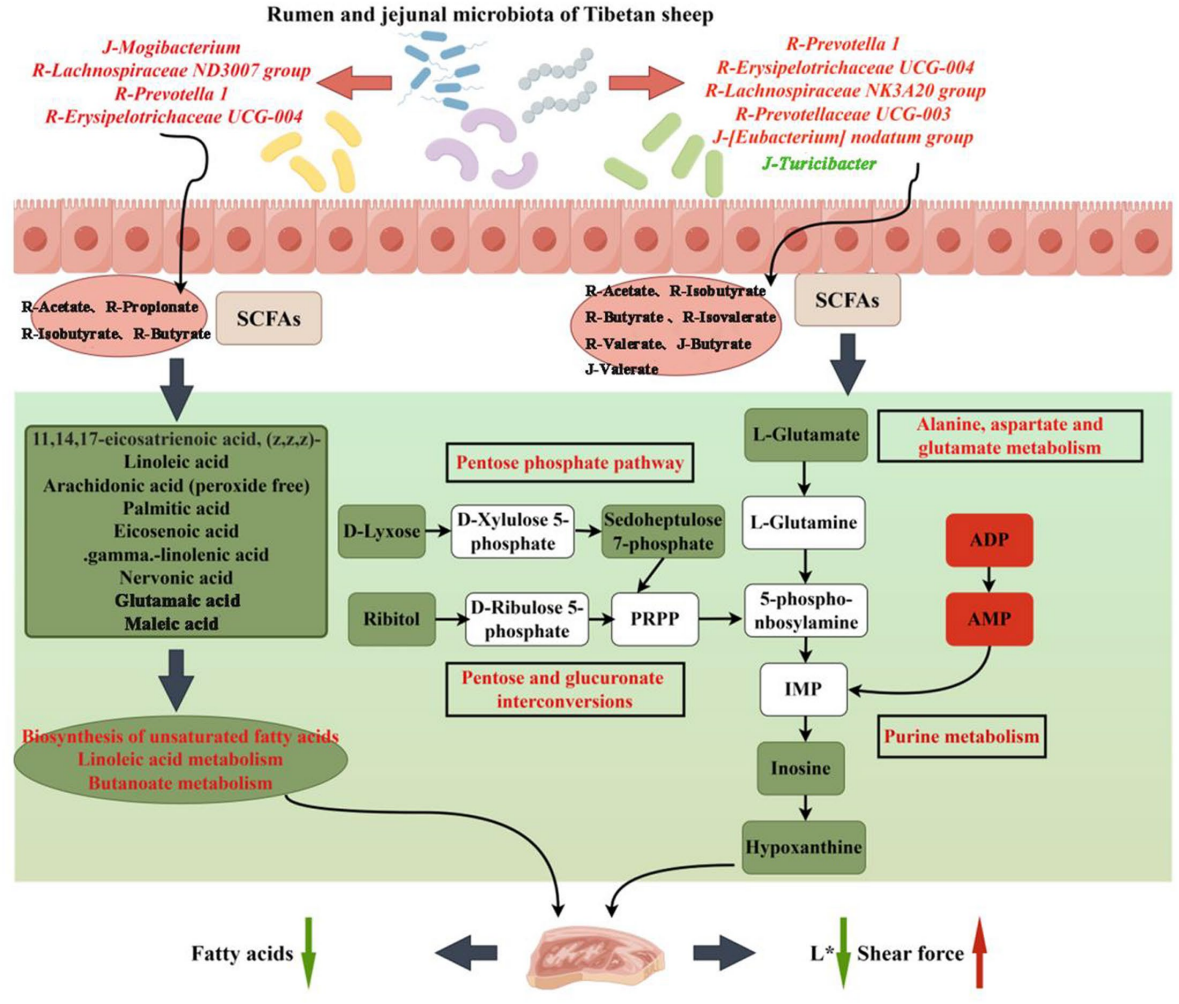
Hypothetical pathways and potential mechanisms related to the gastrointestinal flora, muscle metabolites and meat quality changes in RPSAA Tibetan sheep. The green and red boxes indicate the down-regulation and up-regulation of metabolites, respectively; The green and red arrows indicate an increase or decrease of the meat quality index.

The composition and fatty acids content of meat not only affect its flavor but also affect human health. For example, research has shown that PUFA possessed anti-obesity and anti-inflammatory properties. Similarly, the intake of n-3 and n-6 PUFA is essential for normal human growth and development due to their regulatory role in cell functions, signal transduction and immune response (Liu et al., 2023). Of these, C20:5 N3 (EPA), C22:6 N3 (DHA) and C22:5 N3 (DPA) are particularly important n-3 PUFAs as they are functional fatty acids which are involved in regulating cholesterol, while reducing risks of human neurodegeneration, coronary heart disease and thrombosis (Montenegro et al., 2022). Similarly, n-6 PUFA such as arachidonic acid (AA) and linoleic acid (LA) are of significance as an increase in LA intake can improve plasma lipids, blood glucose control and insulin resistance (Marangoni et al., 2020). This work applied GC–MS to determine the composition and fatty acids content of three groups of Tibetan sheep meat and found that the n-3 PUFA, EPA, DHA, DPA and C20:3 N6 content in AP-2 significantly decreased. In fact, the overall fatty acids content also showed a downward trend. Non-target metabolomics detection further showed that fatty acids such as linoleic acid, arachidonic acid, palmitic acid, γ-linolenic acid and eicosenoic acid were downregulated in AP-2 (AP-2 vs. AP-1), with their involvement in the biosynthesis of unsaturated fatty acids, linoleic acid metabolism and butanoate metabolism also downregulated (Table 4). In contrast, the biosynthesis of unsaturated fatty acids and linoleic acid metabolism (AP-3 vs. AP-2) in AP-3 were upregulated, with the amount of γ-linolenic acid and linoleic acid also increased (Table 4). Research has found that butanoate metabolism, through the β-Hydroxyl group-β-Methylglutaryl CoA pathway, increased lipid synthesis (Liu et al., 2018). However, due to the significant correlation between these fatty acids, metabolites and the microbiota in the rumen and jejunum (Figure 7B), it was speculated that in AP-2, the downregulation of pathways related to lipid synthesis, and hence a decrease in the fatty acids content, was caused by changes in the gastrointestinal microbiota of Tibetan sheep (Figure 8). These results were also reflected in the works of Wen et al. and Conde-Aguilera et al. who found that the addition of sufficient MET to diets resulted in a lower fat content in chicken breast compared to the MET-deficient group (Conde-Aguilera et al., 2010; Wen et al., 2017). This observation could be attributed to a MET-induced increase in carnitine content that not only promoted the oxidation of fatty acids but also reduced the amount of constant fatty acids that could be stored in adipose tissues (Zhan et al., 2006). Finally, being precursors of flavor substances, fatty acids can influence the flavor of meat through reactions such as lipid oxidation. A decrease in the fatty acids content, as it was the case for AP-2, would therefore affect its flavor.

Overall, compared with the ordinary amino acid group, the WHC and texture of the groups fed with RPSAA increased, resulting in better food quality. In addition, when comparing the two groups of RPSAA, the fatty acid metabolism pathway was significantly upregulated in AP-3. This could be because, in AP-2, the endogenous CYS synthesized by MET through the sulfur transfer pathway was insufficient to meet the needs of Tibetan sheep. On the other hand, the exogenous CYS added to feed in AP-3 helped to meet the dietary requirements, hence increasing the production of antioxidant substances such as glutathione and taurine, and preventing the oxidation of fatty acids (Medina et al., 2022). As a result, AP-2 has the best edible quality, but its fatty acid nutrition is reduced.

Amino acids are immediately decomposed by microorganisms in the rumen to produce ammonia. In this case, RPAA can reduce the degradation within the rumen, thereby allowing the amino acids to enter the small intestine. This not only improves the utilization rate of amino acids but also increases animal production (Ma et al., 2021; Liu et al., 2023). However, RPAA decomposed in the rumen and entering the small intestine may also alter the gastrointestinal microbiota. As such, this decomposition process can influence fermentation parameters and even regulate some metabolism in animals (Zhang et al., 2022a; Liu et al., 2023; Wei et al., 2023). Previous studies found that the addition of RPLYS and RPSAA did not significantly affect the rumen pH, ammoniacal nitrogen and total concentration of SCFAs in yaks (Liu et al., 2023) and brown lambs (Liu et al., 2021), with these findings being consistent with those of the current study. However, the ammonia-nitrogen concentration in the jejunum of the RPSAA group significantly decreased, while the concentrations of butyric acid and valeric acid significantly increased, thereby indicating that RPSAA improved the utilization rate of AA in the jejunum and promoted jejunal fermentation (Wang et al., 2022) This could also explain the improved carcass quality in both RPSAA groups.

Gastrointestinal microbiota plays a crucial role in the nutrition and health of ruminants. As far as RPSAA is concerned, *Bacteroidetes* and *Proteobacteria* are particularly affected within the rumen. In this study, the three groups did not differ significantly in terms of their relative proportion of the top five main phylum in the jejunum. *Bacteroidetes*, the largest portal in the rumen, is responsible for protein hydrolysis, carbohydrate degradation and the fermentation of amino acids into acetate (Hinsu et al., 2017). On the other hand, *Firmicutes*, the largest portal in the jejunum. Play an important role in the degradation of fiber and cellulose (Crisol-Martínez et al., 2017). The structure of the two is different at the phylum level, with differences also noted in their fermentation modes. Correlation analysis was performed on the microbial communities and fermentation parameters of the rumen and jejunum, with the results showing a close connection between the rumen and jejunum. *Prevotelaceae_ UCG-003* uses various sugars to produce acetic acid and succinic acid while enhancing fiber digestion (Li et al., 2019). *Lachnospiraceae_ NK3A20* can alleviate intestinal inflammation by producing butyric acid to inhibit the production of pro-inflammatory cytokines by intestinal neutrophils (Li et al., 2021). Hence, it is also referred to as a probiotic. Finally, *Turicibacter* is a pathogen that is positively correlated with colitis (Rettedal et al., 2009). The abundance of *Prevotellaceae_ UCG-003* and *Lachnospiraceae_NK3A20* increased in the AP-2 and AP-3 groups, especially with the addition of RPCYS (AP-3 group), while the proportion of *Turicibacter* decreased significantly in the two groups. The first two bacterial genera are positively correlated with butyric acid, isobutyric acid, valeric acid, isovaleric acid and acetic acid, but negatively correlated with the *Turicibacter* in the jejunum. Therefore, feeding RPAA can be useful to increase the abundance of beneficial bacteria and SCFAs in the rumen of Tibetan sheep. As it flows from the rumen to the jejunum, it further inhibits the production of harmful bacteria and maintains the healthy development of the jejunum. The influence of diet on gastrointestinal microbiota can also regulate muscle metabolites and affect meat quality (Peng et al., 1973). In this study, the abundance of microbiota in the rumen and jejunum was closely related to the amount of fatty acids and purine metabolism as well as to the L^*^ and SF of the LL meat. Therefore, it was speculated that feeding RPSAA promotes fermentation in the rumen and jejunum of Tibetan sheep, thereby affecting fatty acids and purine metabolism in the LL, and ultimately altering L^*^ and SF (Figure 8). However, these changes in meat quality may also be caused by the complex transformation relationship between MET, cysteine, and CYS, leading to metabolic changes in Tibetan sheep and synergistic effects on their living environment. As noted before, further research would be required to ascertain the above speculation, and especially to verify the specific mechanism through which rumen and jejunal microbiota influence the quality of Tibetan sheep meat.

## Conclusion

A number of studies have shown that reducing the level of crude proteins in feed and adding RPAA can reduce the emission of nitrogenous pollutants from animals without affecting their growth. In this study, feeding the RPMET group (AP-2) improved the carcass quality of Tibetan sheep by increasing their carcass weight, abdominal fat, and back fat; Compared with the AP-3 group, the AP-2 group achieved the best edible quality by reducing brightness, cooking loss, and shear force, but the nutritional value of fatty acids in the AP-2 group decreased. Further analysis indicates that the addition of RPSAA affects important metabolic pathways and metabolites in Tibetan sheep muscles. The downregulation of unsaturated fatty acid biosynthesis, linoleic acid metabolism, and butanoate metabolism in AP-2 could have been responsible for the decrease in the fatty acids content. Finally, correlation analysis showed that the increase of beneficial microbiota (*Prevotella 1, Lachnospiraceae NK3A20 group, Prevotella UCG-003, Lachnospiraceae ND3007 group*) in the rumen of AP-2 and AP-3 could increase the abundance of beneficial microbiota (*Eubacterium nodatum group, Mogibacterium group*) in the jejunum and inhibit the growth of harmful ones (*Turicibacter*). In addition, an increase in the abundance of gastrointestinal microbiota and SCFAs also influenced the key metabolites of LL (inosine, hypoxantine, AMP, ADP, L-glutamate, etc.), thereby regulating purine metabolism and ultimately affecting the brightness and tenderness of LL. In a word, the results suggested that RPAA can improve the overall quality of Tibetan sheep’s meat.

## Data availability statement

The datasets presented in this study can be found in online repositories. The names of the repository/repositories and accession number(s) can be found in the article/Supplementary material.

## Ethics statement

The animal studies were approved by Institutional Animal Care and Use Committee Guidelines of Qinghai University (protocol number 0515). The studies were conducted in accordance with the local legislation and institutional requirements. Written informed consent was obtained from the owners for the participation of their animals in this study.

## Author contributions

JL: Conceptualization, Data curation, Formal analysis, Methodology, Software, Validation, Visualization, Writing – original draft. LH: Conceptualization, Formal analysis, Funding acquisition, Methodology, Project administration, Supervision, Visualization, Writing – original draft, Writing – review & editing. SH: Conceptualization, Investigation, Resources, Validation, Writing – review & editing. LG: Formal analysis, Software, Writing – review & editing. ZY: Data curation, Writing – review & editing. SS: Software, Writing – review & editing. ZW: Investigation, Writing – review & editing. BY: Investigation, Writing – review & editing.
