# Supplementary material for: Integrated metabolome and microbiome analysis reveals the effect of rumen-protected sulfur-containing amino acids on the meat quality of Tibetan sheep meat
Source: Front Microbiol. 2024 Feb 8;15:1345388. doi: 10.3389/fmicb.2024.1345388 (PMC10883651; doi:10.3389/fmicb.2024.1345388)
Supplement: Supplementary file 1 [file Data_Sheet_1.docx]

# 1 Supplementary Figures and Tables

**1.1 Supplementary Figures**


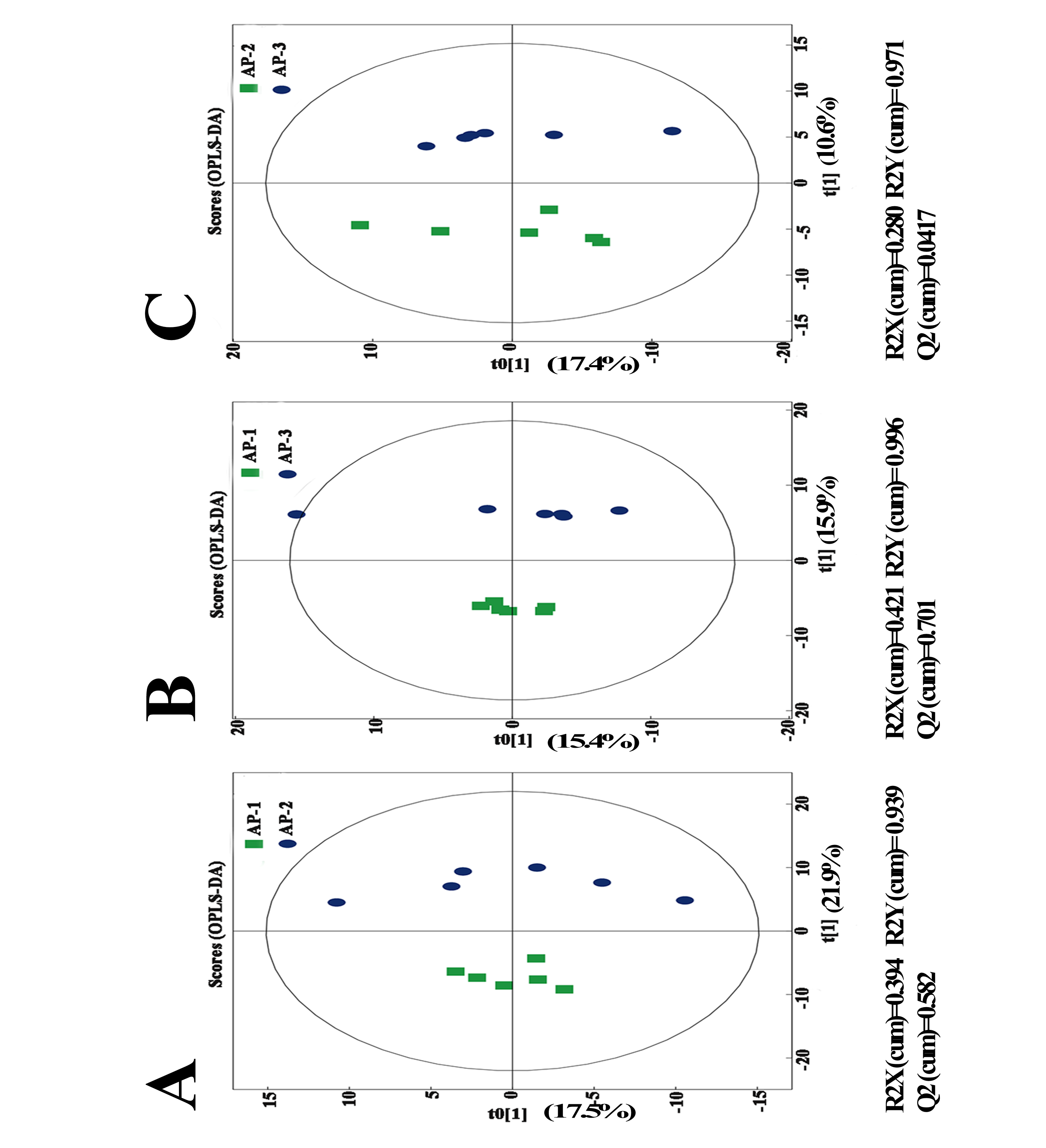


**Supplementary Figure 1.** A (AP-1 and AP-2), B (AP-1 and AP-3), and c (AP-2 and AP-3) are the OPLS-DA scores of three groups of samples in the negative ion detection mode were compared in pairs.


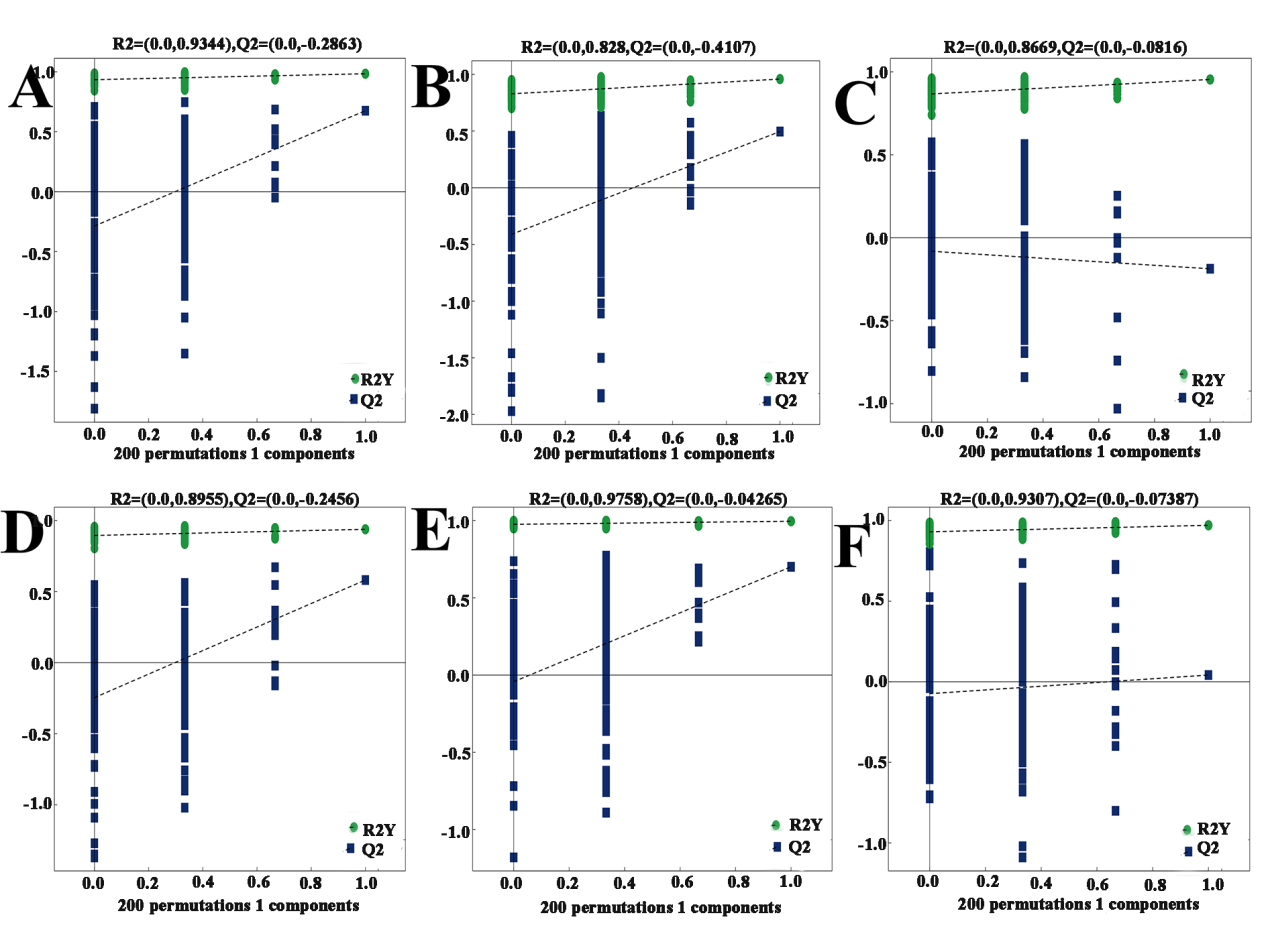


**Supplementary Figure 2.** OPLS-DA permutation test chart for pairwise comparison of three groups in positive and negative ion mode, A (AP-1 and AP-2), B (AP-1 and AP-3), C (AP-2 and AP-3) are in the positive ion mode; D (AP-1 and AP-2),E(AP-1 and AP-3), F (AP-2 and AP-3) are in negative ion mode.

**1.2 Supplementary Tables**

**Supplementary Table 1.** Dietary ingredients and nutritional level of diets (air-dry matter basis, %)

| Items | AP-1group（%） | AP-2group（%） | AP-3group（%） |
| --- | --- | --- | --- |
| Dietary ingredients（% DM） |  |  |  |
| corn | 42.28 | 42.28 | 42.28 |
| Soybean meal | 2.45 | 2.45 | 2.45 |
| Rapeseed meal | 8.00 | 8.00 | 8.00 |
| Cottonseed meal | 16.00 | 16.00 | 16.00 |
| Oat silage | 2.00 | 2.00 | 2.00 |
| Oaten hay | 2.00 | 2.00 | 2.00 |
| Mineral salt | 12.00 | 12.00 | 12.00 |
| limestone | 1.00 | 1.00 | 1.00 |
| baking soda | 1.00 | 1.00 | 1.00 |
| Dicalcium phosphate | 0.10 | 0.10 | 0.10 |
| Mineral/Vitamin Premix | 0.60 | 0.60 | 0.60 |
| Total | 100.00 | 100.00 | 100.00 |
| Nutritional levels (% DM） |  |  |  |
| Digestive energy (MJ/kg) | 11.08 | 11.08 | 11.08 |
| crude protein | 13.25 | 13.25 | 13.25 |
| Coarse ash content | 4.53 | 4.53 | 4.53 |
| crude fat | 5.13 | 5.13 | 5.13 |
| Neutral washing fiber | 26.38 | 26.38 | 26.38 |
| Acid washing fiber | 16.99 | 16.99 | 16.99 |
| calcium | 0.87 | 0.87 | 0.87 |
| phosphorus | 1.02 | 1.02 | 1.02 |
| Add to premix | Add 3kg of regular LYS to 1 ton: 1kg of regular MET | Add 1.5kg RPLYS to 1 ton: 0.5kg RPMET | 1 ton added with 1.5kg RPLYS: 0.25kg RPMET: 0.25kg RPCYS |

**Supplementary Table 2.** Fatty acid standard curve results

| Name | Linear equation | R^2 | Linear range (ug/ml) |
| --- | --- | --- | --- |
| C4:0 | y=0.066680*x+0.000903955 | 0.9987 | 0.002-50 |
| C6:0 | y=0.266594*x+0.000002371602 | 0.9999 | 0.002-50 |
| C8:0 | y=0.371119*x+0.000004201324 | 0.9998 | 0.002-50 |
| C10:0 | y=0.241341*x+0.009446 | 0.9974 | 0.004-100 |
| C11:0 | y=0.318213*x+0.0001589773 | 0.9991 | 0.002-50 |
| C12:0 | y=0.257171*x+0.009181 | 0.9989 | 0.004-100 |
| C13:0 | y=0.268695*x+0.001147 | 0.9988 | 0.002-50 |
| C14:0 | y=0.225594*x+0.009428 | 0.998 | 0.002-50 |
| C14:1N5 | y=0.072335*x+0.002819 | 0.9982 | 0.002-50 |
| C15:0 | y=0.221815*x+0.009442 | 0.9979 | 0.002-50 |
| C15:1N5 | y=0.076850*x+0.002892 | 0.9984 | 0.002-50 |
| C16:0 | y=0.200059*x+0.069542 | 0.9978 | 0.004-100 |
| C16:1N7 | y=0.060288*x+0.002086 | 0.9986 | 0.002-50 |
| C17:0 | y=0.200807*x+0.046396 | 0.9973 | 0.004-100 |
| C17:1N7 | y=0.063949*x+0.007322 | 0.999 | 0.002-50 |
| C18:0 | y=0.202926*x+0.020104 | 0.9973 | 0.004-100 |
| C18:1TN9 | y=0.058658*x+0.001985 | 0.9987 | 0.002-50 |
| C18:1N9 | y=0.056265*x+0.005142 | 0.9976 | 0.004-100 |
| C18:2TTN6 | y=0.073651*x+0.002253 | 0.9989 | 0.002-50 |
| C18:2N6 | y=0.074869*x+0.002333 | 0.9989 | 0.002-50 |
| C18:3N6 | y=0.076810*x+0.0003154531 | 0.9992 | 0.004-100 |
| C18:3N3 | y=0.085137*x+0.002608 | 0.9988 | 0.002-50 |
| C20:0 | y=0.190836*x+0.017724 | 0.9975 | 0.004-100 |
| C20:1N9 | y=0.062761*x+0.002012 | 0.9988 | 0.002-50 |
| C20:2N6 | y=0.071695*x+0.002223 | 0.9989 | 0.002-50 |
| C21:0 | y=0.269985*x+0.0009428574 | 1 | 0.002-50 |
| C20:3N6 | y=0.072340*x+0.0007136302 | 0.9999 | 0.002-50 |
| C20:4N6 | y=0.067336*x+0.001909 | 0.9991 | 0.002-50 |
| C20:3N3 | y=0.095307*x+0.0002186293 | 0.9989 | 0.002-50 |
| C22:0 | y=0.243074*x+0.0002115054 | 0.9995 | 0.004-100 |
| C20:5N3 | y=0.077635*x+0.002756 | 0.9986 | 0.002-50 |
| C22:1N9 | y=0.062700*x+0.001708 | 0.9991 | 0.002-50 |
| C22:2N6 | y=0.074003*x+0.0002638267 | 0.9999 | 0.002-50 |
| C23:0 | y=0.222361*x+0.00005008438 | 0.9999 | 0.002-50 |
| C22:4N6 | y=0.072347*x+0.0003255536 | 0.9999 | 0.002-50 |
| C22:5N6 | y=0.068060*x+0.0004308835 | 0.9997 | 0.002-50 |
| C24:0 | y=0.196410*x+0.0002729661 | 0.9997 | 0.004-100 |
| C22:5N3 | y=0.073322*x+0.00394 | 0.9995 | 0.002-50 |
| C24:1N9 | y=0.091834*x+0.0002070603 | 0.9993 | 0.002-50 |
| C22:6N3 | y=0.080596*x+0.0004494154 | 0.9997 | 0.002-50 |

**Supplementary Table 3.** Effects of RPSAAs on amino acids in the *longissimus lumborum* muscle of Tibetan Sheep (umol/g tissue )

|  | AP-1 | AP-2 | AP-3 |
| --- | --- | --- | --- |
| glutamate | 0.37±0.08 | 0.16±0.04 | 0.17±0.05 |
| glycine | 2.81±0.15 | 2.82±0.17 | 2.52±0.09 |
| lysine | 0.39±0.04 | 0.39±0.04 | 0.36±0.01 |
| aspartic acid | 0.02±0.00 | 0.03±0.02 | 0.03±0.01 |
| arginine | 0.35±0.04 | 0.43±0.01 | 0.41±0.05 |
| serine | 0.42±0.03 | 0.45±0.02 | 0.41±0.03 |
| methionine | 0.10±0.01 | 0.11±0.01 | 0.10±0.00 |
| phenylalanine | 0.26±0.03 | 0.26±0.01 | 0.24±0.01 |
| tyrosine | 0.23±0.02 | 0.24±0.00 | 0.21±0.01 |
| leucine | 0.38±0.04 | 0.43±0.01 | 0.37±0.01 |
| isoleucine | 0.20±0.03 | 0.24±0.01 | 0.21±0.01 |
| histidine | 1.39±0.08 | 1.24±0.04 | 1.41±0.02 |
| proline | 0.24±0.02 | 0.24±0.03 | 0.23±0.01 |
| valine | 0.40±0.04 | 0.45±0.02 | 0.43±0.02 |
| threonine | 0.36±0.04 | 0.39±0.03 | 0.36±0.01 |
| alanine | 4.59±0.27 | 4.70±0.19 | 4.92±0.15 |
| asparagine | 0.17±0.01 | 0.20±0.01 | 0.20±0.01 |
| creatine | 9.31±0.04 | 9.19±0.28 | 9.24±0.32 |
| citrulline | 0.13±0.02 | 0.08±0.01 | 0.09±0.02 |
| glutamine | 2.78±0.63 | 2.96±0.08 | 2.51±0.17 |
| cysteine | 0.01±0.00 | 0.02±0.02 | 0.00±0.00 |
| creatinine | 0.13±0.00^a^ | 0.11±0.00^b^ | 0.11±0.0^b^ |
| tryptophan | 0.32±0.03 | 0.34±0.03 | 0.31±0.01 |
| hydroxyproline | 0.09±0.01 | 0.08±0.01 | 0.07±0.00 |
| ornithine | 0.04±0.01 | 0.04±0.00 | 0.03±0.00 |
| taurine | 7.09±0.74 | 7.92±0.61 | 7.93±0.37 |
| cystine | 0.63±0.06 | 0.61±0.06 | 0.60±0.05 |
| choline | 4.91±1.28 | 6.67±1.26 | 4.93±0.69 |
| aminoadipic acid | 0.02±0.00^b^ | 0.05±0.01^a^ | 0.03±0.00^b^ |
| FAAs | 3.55±0.40 | 3.44±0.37 | 3.12±0.19 |
| SAAs | 8.81±0.82 | 8.99±0.57 | 8.79±0.29 |
| BAAs | 3.63±0.45 | 3.73±0.13 | 3.68±0.21 |
| EAAs | 2.41±0.40 | 2.60±0.17 | 2.37±0.09 |
| NEAAs | 10.43±0.79 | 10.32±0.67 | 10.30±0.24 |
| TAAs | 38.16±4.58 | 40.85±2.31 | 38.40±1.87 |

^a, b, c^ means that different letters on the same line indicate statistically significant differences (*P<0.05*); the same letter means no significant difference (*P>0.05*). FAAs: Flavor amino acids; SAAs: Sweet amino acids; BAAs: Bitter amino acids; EAAs: Essential amino acids; NEAAs: Nonessential amino acids;TAAs: Total amino acids

**Supplementary Table 4.** Effects of RPSAAs on fatty acids in the *longissimus lumborum* muscle of Tibetan Sheep (ug/g tissue)

| Item | AP-1 | AP-2 | AP-3 |
| --- | --- | --- | --- |
| C10:0 | 0.61±0.00 | 0.19±0.06 | 1.39±0.66 |
| C11:0 | 0.01±0.00 | 0.01±0.00 | 0.02±0.01 |
| C12:0 | 1.35±0.72 | 0.55±0.08 | 2.61±1.56 |
| C13:0 | 0.06±0.04 | 0.02±0.00 | 0.10±0.06 |
| C14:0 | 39.53±16.05 | 18.49±1.31 | 40.94±15.84 |
| C14:1N5 | 1.76±0.89 | 0.72±0.04 | 1.80±0.79 |
| C15:0 | 3.55±1.73 | 1.67±0.06 | 4.12±1.70 |
| C15:1N5 | 0.71±0.32 | 0.24±0.04 | 0.90±0.43 |
| C16:0 | 314.29±99.04 | 178.94±13.44 | 290.12±67.03 |
| C16:1N7 | 42.06±16.81 | 23.14±3.82 | 37.47±8.63 |
| C17:0 | 11.84±5.43 | 6.94±0.87 | 14.18±5.02 |
| C17:1N7 | 11.21±3.60 | 7.62±0.74 | 12.13±3.50 |
| C18:0 | 199.06±78.74 | 115.48±20.25 | 195.16±51.33 |
| C18:1TN9 | 137.98±55.74 | 78.19±13.16 | 135.01±36.78 |
| C18:1N9 | 532.07±171.38 | 338.08±54.01 | 515.61±90.78 |
| C18:2N6 | 58.06±10.86 | 32.64±0.30 | 53.34±12.79 |
| C18:3N6 | 1.09±0.25 | 0.65±0.01 | 0.97±0.21 |
| C18:3N3 | 6.30±2.12 | 2.66±0.12 | 5.43±1.96 |
| C20:0 | 1.38±0.78 | 0.42±0.11 | 1.10±0.30 |
| C20:1N9 | 4.16±2.02 | 1.19±0.15 | 2.64±0.74 |
| C20:2N6 | 4.43±0.80 | 3.84±0.26 | 4.50±0.60 |
| C21:0 | 0.04±0.02 | 0.02±0.00 | 0.04±0.00 |
| C20:3N6 | 2.22±0.09^a^ | 1.56±0.05^b^ | 1.93±0.12^a^ |
| C20:4N6 | 25.10±1.96 | 19.99±1.57 | 23.00±2.81 |
| C20:3N3 | 0.33±0.06 | 0.24±0.02 | 0.33±0.04 |
| C22:0 | 0.22±0.05 | 0.13±0.01 | 0.17±0.00 |
| C20:5N3 | 3.48±0.44^a^ | 2.20±0.18^b^ | 2.30±0.06^b^ |
| C22:1N9 | 1.64±0.85 | 0.45±0.03 | 0.95±0.28 |
| C22:2N6 | 0.39±0.08 | 0.37±0.07 | 0.36±0.10 |
| C23:0 | 0.01±0.01 | 0.01±0.00 | 0.02±0.00 |
| C22:4N6 | 1.83±0.05 | 1.52±0.13 | 1.66±0.20 |
| C22:5N6 | 0.60±0.08 | 0.49±0.04 | 0.45±0.07 |
| C24:0 | 0.03±0.01 | 0.01±0.00 | 0.03±0.00 |
| C22:5N3 | 5.53±0.33^a^ | 4.05±0.51^b^ | 5.15±0.27^ab^ |
| C24:1N9 | 0.24±0.12 | 0.13±0.03 | 0.24±0.06 |
| C22:6N3 | 1.07±0.03^a^ | 0.73±0.15^b^ | 0.94±0.05^ab^ |
| SFAs | 571.99±202.08 | 322.89±35.16 | 550.00±142.29 |
| MUFAs | 731.82±251.57 | 449.77±71.79 | 706.76±141.14 |
| PUFAs | 110.44±15.14 | 70.94±1.77 | 100.35±15.25 |
| n-3 PUFAs | 16.71±2.29^a^ | 9.88±0.96^b^ | 14.14±2.10^ab^ |
| n-6 PUFAs | 93.73±13.01 | 61.06±1.60 | 86.21±13.21 |

^a, b, c^ means that different letters on the same line indicate statistically significant differences (*P<0.05*); the same letter means no significant difference (*P>0.05*). SFAs：Saturated fatty acids； MUFAs: Monounsaturated fatty acids; PUFA: Polyunsaturated fatty acids.

**Supplementary Table 5.** Evaluation parameters of OPLS-DA models in the positive and negitive ion mode.

|  | R2X (cum) | R2Y (cum) | Q2 (cum) |
| --- | --- | --- | --- |
| positive |  |  |  |
| AP-1 vs AP-2 | 0.578 | 0.984 | 0.676 |
| AP-1 vs AP-3 | 0.279 | 0.958 | 0.495 |
| AP-2 vs AP-3 | 0.356 | 0.954 | -0.187 |
| negitive |  |  |  |
| AP-1 vs AP-2 | 0.394 | 0.939 | 0.582 |
| AP-1 vs AP-3 | 0.421 | 0.996 | 0.701 |
| AP-2 vs AP-3 | 0.280 | 0.971 | 0.0417 |

**Supplementary Table 6.** Effect of feeding RPSAA on alpha diversity of rumen bacteria of Tibetan sheep.

|  | AP-1 | AP-2 | AP-3 |
| --- | --- | --- | --- |
| shannon | 5.55±0.60^b^ | 6.24±0.42^a^ | 5.98±0.14^ab^ |
| simpson | 0.88±0.06^b^ | 0.95±0.03^a^ | 0.93±0.02^ab^ |
| ace | 1239.26±303.86 | 1190.20±210.80 | 1287.79±221.58 |
| chao1 | 1196.49±292.03 | 1161.57±195.19 | 1255.24±211.77 |

^a, b, c^ means that different letters on the same line indicate statistically significant differences (*P<0.05*); the same letter means no significant difference (*P>0.05*).

**Supplementary Table 7.** Effect of feeding RPSAA on alpha diversity of jejunum bacteria of Tibetan sheep.

|  | AP-1 | AP-2 | AP-3 |
| --- | --- | --- | --- |
| shannon | 5.67±0.60 | 5.14±0.58 | 4.87±0.80 |
| simpson | 0.87±0.04 | 0.87±.05 | 0.81±0.12 |
| ace | 1631.06±229.94 | 1176.20±150.73 | 1200.91±117.46 |
| chao1 | 1590.82±228.87 | 1143.47±147.25 | 1159.91±119.36 |

^a, b, c^ means that different letters on the same line indicate statistically significant differences (*P<0.05*); the same letter means no significant difference (*P>0.05*).
